# Dietary Polyphenols (Flavonoids) Derived from Plants for Use in Therapeutic Health: Antioxidant Performance, ROS, Molecular Mechanisms, and Bioavailability Limitations

**DOI:** 10.3390/ijms27031404

**Published:** 2026-01-30

**Authors:** Tomas Gabriel Bas

**Affiliations:** Escuela de Ciencias Empresariales, Universidad Catolica del Norte, Coquimbno 1780000, Chile; tomas.bas@ucn.cl

**Keywords:** polyphenols, flavonoids, oxidative stress, antioxidants, chronic diseases, functional foods, ROS, nutraceuticals, bioavailability, Nrf2

## Abstract

Plant polyphenols, particularly flavonoids, are prominent bioactives in preventive/complementary therapeutic strategies. This article analyzes how some polyphenols can mitigate oxidative stress and inflammation. These processes are involved in cardiovascular disease, cancer, neurodegeneration, and metabolic disorders. Polyphenols are explored through the integration of direct antioxidant chemistry (radical scavenging via hydrogen atom transfer/single-electron transfer/metal chelation), redox signaling (Keap1–Nrf2/ARE and inflammatory pathways), endogenous antioxidant enzyme systems, and mitochondrial quality control. Unlike previous descriptive reviews, a novel aspect of this manuscript is its evidence-based synthesis, fully supported by structured summary tables that explicitly detail limitations, contradictions, and context dependencies in in vitro, in vivo, and human studies, and identify clinically interpretable endpoints for their application. We describe relevant flavonoids and dietary sources, along with functional outcomes in cardiometabolic–cognitive/neuroprotective–immunometabolic contexts. We integrate representative clinical interventions and nutraceutical applications, highlighting where reported benefits are supported and where the evidence is preliminary. Bioavailability, microbiota-driven biotransformation, and dose realism are considered the primary determinants of in vivo relevance, rather than secondary or descriptive considerations. Future research should prioritize standardized exposure and metabolite profile, dose-appropriate interventions, harmonized clinical endpoints, and stratification strategies that account for microbiome-driven interindividual variability to improve reproducibility and inform nutraceutical and therapeutic use.

## 1. Introduction

Plant-derived bioactive compounds, widely distributed in fruits, vegetables, and oils, contribute to significant advances in both preventive biomedicine and various therapeutic health strategies. Among the most important compounds are polyphenols, which have a heterogeneous chemical structure that, consequently, determines their biological activity, bioavailability, and potential therapeutic efficacy [1,2,3,4]. These compounds are characterized by the presence of numerous phenolic hydroxyl groups attached to aromatic ring structures and are synthesized in plants primarily through the shikimate and phenylpropanoid pathways, conferring their distinctive chemical and biological properties [5,6,7,8]. Numerous subclasses of polyphenols exist, including flavonoids, phenolic acids, stilbenes, lignans, and tannins, which have different properties due to their potential to modulate cellular processes involved in inflammation, apoptosis, energy metabolism, and aging [9]. These bioactive compounds have gradually been integrated into the manufacture of functional foods or nutraceuticals. Studies also highlight them as an evidence-based alternative to the Mediterranean diet and other dietary patterns associated with increased longevity and a lower incidence of chronic diseases [10]. However, the complexity of polyphenol chemistry, combined with their diverse biological activities, requires a comprehensive understanding of their molecular structures, dietary sources, and extraction methodologies to optimize their bioavailability and therapeutic applications in current clinical medicine [11].

The antioxidant capacity of polyphenols (flavonoids) is particularly notable against reactive oxygen species (ROS) [12,13]. ROS are molecules that, as their name suggests, contain oxygen, which is highly reactive and whose unpaired electrons are crucial for cell signaling and homeostasis [12]. ROS include both radical and non-radical species. The former possess unpaired electrons, such as superoxide and hydroxyl radicals, while the latter include hydrogen peroxide and singlet oxygen [13,14]. The balance between ROS production and antioxidant defenses is vital, since excess ROS can cause oxidative stress and damage essential biomolecules, such as DNA, proteins, and lipids, thus contributing to various pathologies such as premature aging, cancer, neurodegenerative disorders, inflammation, and mitochondrial homeostasis [15,16].

The synthesis of the mechanism related to the antioxidant capacity of flavonoids could be quantified using assays such as FRAP and ABTS, providing information on their potential health benefits [17]. However, it is essential to highlight two aspects of flavonoid functionality:

(i) Chemical antioxidant capacity, which refers to its ability to directly eliminate free radicals;
(ii) Modulation of cellular pathways, where polyphenols activate specific signaling pathways, inducing protective mechanisms in cells [18]. Chemical antioxidant mechanisms primarily involve electron donation to neutralize ROS [19]. Some flavonoids, such as quercetin, have been extensively studied for their antioxidant properties and their ability to interact with various biological molecules, which could influence cellular redox states [20]. Meanwhile, modulation of cellular pathways often extends beyond immediate antioxidant actions, potentially affecting long-term cellular conditions such as inflammatory responses and metabolic adaptations [21]. This can be observed through contributions to intracellular signaling pathways via sirtuins in adipose tissue, where polyphenols stimulate the darkening process and thus enhance thermogenesis [22].

However, to confirm these mechanisms, more clinical evidence is needed to link theoretical benefits with practical health outcomes. The clinical application of these findings depends on understanding not only the chemical properties but also the biological impacts of polyphenol-rich interventions through specifically designed trials. This would include evaluating intervention strategies that use high concentrations of polyphenols to determine their efficacy in the prevention and management of specific diseases [23,24]. This pathological mechanism gives clinical and therapeutic relevance to plant bioactive compounds capable of combating ROS and modulating the expression of antioxidant enzymes. In this regard, superoxide dismutase (SOD) converts the superoxide radical into peroxide, and glutathione peroxidase (GPX) and catalase break it down into water and oxygen, thus protecting the mitochondrial integrity of the organism through signaling pathways such as Nrf2 [25].

Under oxidative/electrophilic stress, the disruption of Nrf2 degradation mediated by the Keap1 protein allows Nrf2 to accumulate and translocate to the cell nucleus, where it binds to antioxidant response elements (AREs) and induces cytoprotective genes [26]. In the nucleus, Nrf2 activates genes related to antioxidant responses, including those that help produce protective enzymes such as glutamate–cysteine ligase (GCLC) and NADPH: quinone oxidoreductase 1 (NQO1) [27]. These enzymes enhance the ability of the cell to manage oxidative damage and optimize the overall antioxidant capacity, which can vary depending on cell type and circumstances [28]. This mechanism of Nrf2 indicates its central role in cellular protection against oxidative stress, as alterations in this pathway can influence inflammation and other pathological conditions [29]. Therefore, Nrf2 not only plays a protective role by activating the expression of antioxidant genes, but also contributes to the regulation of cellular responses to oxidative challenges [30].

Similarly, some advanced analytical technologies, such as Ultra High-Performance Liquid Chromatography coupled with High-Resolution Mass Spectrometry (UPLC-Q-Orbitrap) and High-Resolution Precision Mass Spectrometry (HRMS), have enabled the detailed characterization of these compounds [12]. This facilitates their application in nutrition, pharmacology, cosmetology, and personalized medicine strategies. However, a fundamental element is the quality and bioavailability of antioxidants, which depends not only on the original sources, but also on the techniques used to extract polyphenols from plant materials [31]. In this regard, progress has been made toward innovative approaches that prioritize efficiency, sustainability, high-quality bioavailability, and encapsulation and preservation of these compounds.

It is important to note that although polyphenols (flavonoids) have been extensively studied as antioxidants in the diet, the main novelty and contribution of this review is to provide a comprehensive synthesis that:

(i) Includes direct antioxidant chemistry (HAT/SET and metal chelation) with oxidation–reduction-sensitive signaling (Keap1–Nrf2/ARE and key inflammatory pathways) and mitochondrial quality control processes within a unified mechanistic framework;
(ii) Maps the limitations, contradictions and context dependencies that explain divergent results in in vitro, in vivo, and human studies (including dose realism and metabolite predominance) through summaries focused on the strength of evidence and its respective limitations;
(iii) Consolidates translational constraints (bioavailability, microbiota-driven biotransformation, and diet/drug interactions), along with a synthesis of clinical evidence to support the mechanistic interpretation that is most likely to be clinically relevant;
(iv) Highlights the problems related to nutraceuticals and the importance of regulations;
(v) Analyzes the topic of bioavailability, which considers metabolites as a determinant of in vivo efficacy;
(vi) Examines the relationship between antioxidants and the prevention of chronic diseases (cardiovascular, oncological, neurodegenerative, and immune).

**Figure 1:**
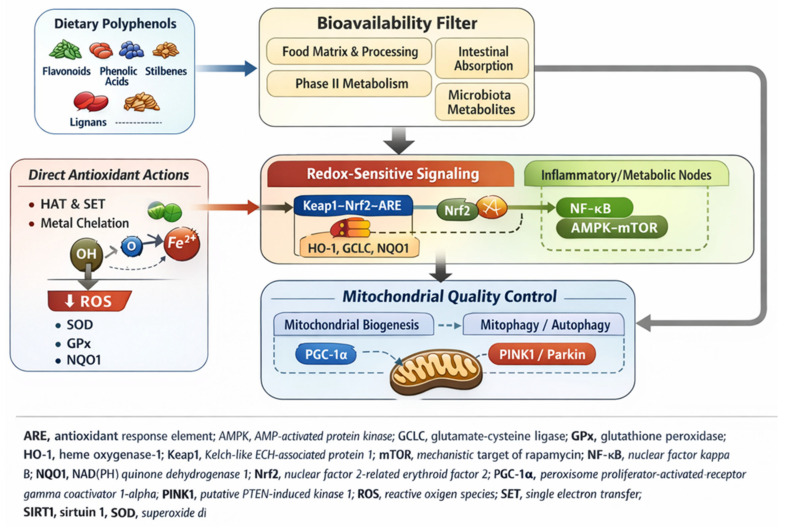
Integrated molecular framework for polyphenol-mediated modulation of oxidative stress.

The novelty is operationalized through explicit synthesis objectives, both in Figure 1 and in the specific summary tables. These guidelines catalog the limitations, contradictions, and context dependencies in the in vitro, in vivo, and human evidence, thus identifying clinically interpretable endpoints and prioritizing future study designs necessary for their application in nutraceuticals and preventive health. A brief overview of the regulations and the importance of comprehensive legislation is also provided, along with an analysis of the value of the growing polyphenol market.

The emphasis was placed on flavonoids because they are the most abundant biocompounds in nature (60% of all known polyphenols).

The following details the different anchor points of the novel findings in this article compared to previous research.

- Integrated mechanistic map unifying redox chemistry → signaling → mitochondrial quality control (Figure 1; Section 2.1).
- Synthesis of the strength of evidence at the mechanistic level with explicit limitations/contradictions (end of Section 2; synthesis table), including assay dependence, supraphysiological dosage, and metabolite predominance.
- Blocks of “limitations/contradictions” in the disease context that separate reported benefits from heterogeneity factors and specify future perspectives (end of Section 3 and Section 4; limitations tables).
- Bioavailability is considered a key determinant of in vivo relevance (Section 5.1, Section 5.2, Section 5.3, Section 5.4 and Section 5.5), integrating microbiota-driven biotransformation and interindividual variability as a key translational axis.
- Structured clinical evidence table across outcome domains, explicitly outlining comparability limits (population, matrix, dose, assessment criteria).
- Explicit mapping of translational constraints (food matrix effects, first-pass metabolism, realism of supplement versus diet dosage, diet–drug interactions/risks of polypharmacy) to maintain a clinically sound mechanistic interpretation (Section 5.3, Section 5.4, Section 5.5 and Section 5.6).
- Encapsulation framed as exposure optimization (Section 5.7) rather than an independent extraction technology—the extract content is removed, while retaining processing variables relevant to exposure (particle size, fermentation).
- Regulatory perspective + nutraceuticals + market implementation (Section 6), linking evidence quality to claims, standardization, and governance.

Regarding the literature search strategy and the eligibility criteria for references, this article reviews in detail 400 bibliographic references obtained from databases such as Scopus, Web of Science, ScienceDirect, Google Scholar, Core Collections, Compendex, Derwent, PubMed/MEDLINE, and Innovation Index. Bibliographic evidence focused primarily on the last four years (2022–2025) to ensure the most current and innovative research related to plant-derived bioactive compounds, using the PRISMA methodology (Preferred Reporting Items for Systematic Reviews and Meta-Analyzes) [32,33]. Search strings combined controlled terms using Boolean operators and truncation strategies. Keywords were primarily related to: “polyphenols”; “flavonoids”; “oxidative stress”; “antioxidants”; “chronic diseases and their specific derivatives” (cardiovascular, cancer, neurodegenerative)”; “functional foods”; “ROS”; “nutraceuticals”; “bioavailability”; “phenolic acid”; “resveratrol”; “lignan”; “oxidative stress”; “reactive oxygen species”; “Nrf2”; “metal chelation”; “encapsulation”; “metabolites”; and “gut microbiota.”

The inclusion criteria were the following:

(i) Original peer-reviewed studies, systematic reviews, or authoritative mechanistic reviews;
(ii) Explicit evaluation of antioxidant mechanisms and/or redox-sensitive signaling;
(iii) Studies that report on the identity of polyphenols (or well-defined extracts) and the experimental context (in vitro, in vivo, or human); and
(iv) Articles in English published within the time period specified in the manuscript (extended when further in-depth work was required to explain the core mechanisms).
(v) Each word was selected for its relevance using “AND” and “OR”.

The exclusion criteria were as follows:

(i) Studies that lacked characterization of polyphenols;
(ii) Purely descriptive antioxidant assays, without mechanistic or biological endpoints;
(iii) Conference abstracts and non-peer-reviewed articles;
(iv) Articles written in a language other than English;
(v) Duplicate records.

The relevance of the titles and abstracts was examined, followed by a comprehensive evaluation. For articles addressing the same mechanism, higher-quality evidence was prioritized (human studies and well-controlled in vivo models, followed by mechanistic cell studies), and conflicting findings were selected to obtain a balanced synthesis.

## 2. Polyphenols: Identification, Chemical Structure, and Classification

Polyphenols are a structurally diverse class of secondary metabolites synthesized in plants as glycosides (linked to sugar molecules), aglycones or conjugated forms (e.g., glycosides, esters and polymers), which can sometimes affect their solubility and bioavailability [34]. A common feature of polyphenols is the presence of one or more aromatic (benzene) rings with hydroxyl (–OH) substituents [35]. The antioxidant activity of polyphenols is primarily due to these hydroxyl groups, which can donate hydrogen atoms to neutralize free radicals, and conjugated ring structures that stabilize the resulting phenoxyl radicals (resonance stabilization) [36]. Certain configurations, such as the orthodihydroxy structure, in the B ring of flavonoids (as in the case of the catechol structure of quercetin or catechins) confer a particularly high capacity for radical scavenging and a metal-chelating potential [37]. Therefore, the chemical architecture of polyphenols (number and position of hydroxyl groups, glycosylation, presence of galloyl groups) determines their antioxidant potential. In this respect, it is worth noting that many polyphenols are pigments (anthocyanins, flavones) that act as a protective shield in the defense of plants against environmental factors (light, temperature, stress) [38].

Polyphenols occur naturally, ranging from simple molecules to highly complex polymers, and are classified into different groups and subgroups according to their chemical structure [3]. Table 1 provides an overview of the classification of the main classes of polyphenols, including their structural characteristics and dietary sources, along with some examples.

### 2.1. Different Molecular Mechanisms of Action of Polyphenols

Polyphenols exert their biological effects through multiple interconnected molecular mechanisms. The main mechanism of action of polyphenols involves the direct scavenging of reactive oxygen species (ROS) and free radicals [73]. Polyphenols possess freely available phenolic hydroxyl groups, which allow them to donate electrons or hydrogen atoms to free radicals, effectively neutralizing oxidative damage [74]. The orthophenolic hydroxyl group structure is readily oxidized into quinone structures with considerable potential for ROS scavenging [75]. Polyphenols modulate multiple molecular targets, such as metal chelation, mitochondrial protection, and anti-inflammatory pathways [76]. Certain polyphenols, such as resveratrol, activate SIRT1, promoting mitophagy and mitochondrial homeostasis [77]. Polyphenols also show concentration-dependent effects, which means that at lower concentrations they can promote cell proliferation, while at higher concentrations they induce apoptosis by activating caspases [78].

However, the extent to which these mitochondrial effects reflect direct target interaction versus subsequent context-dependent signaling remains an active area of research. In particular, resveratrol is frequently cited as a prototypical SIRT1-linked polyphenol. Resveratrol is known to influence mitochondrial dynamics and promote mitophagy via the PINK1/Parkin. However, its activation of SIRT1 appears to be substrate-and assay-dependent, and some studies do not support direct activation of SIRT1 by resveratrol [79]. This has led to ongoing debate about its roles in cellular contexts, suggesting possible indirect mechanisms such as AMPK activation or NAD+ metabolic pathways [80].

However, the effects of resveratrol on mitochondrial quality control are complex. Although some studies report an increase in mitochondrial biogenesis through the PGC-1α axis and mitophagy mediated by PINK1/Parkin [81], discrepancies also arise due to variations in experimental models and dosages [82].

Despite the different investigations related to the different molecular mechanisms of action of polyphenols, in Table 2 some limitations and contradictions related to the evidence of Resveratrol–SIRT1–Mitophagy can be observed.

Table 3 provides an integrated view of how different polyphenol (flavonoid) families interact with signaling pathways, supporting their relevance in the prevention and management of pathologies associated with oxidative stress, inflammation, and tissue damage. The main molecular mechanisms of action of polyphenols are addressed, organized according to the type of biological response and the cellular target involved. First, antioxidant mechanisms are detailed, including both the direct scavenging of reactive oxygen species through the donation of electrons or hydrogen atoms (especially in compounds with catechol motifs, such as hydroxytyrosol) and the activation of SOD, catalase, and glutathione peroxidase via the Nrf2 (Nuclear Factor 2) pathway. Nrf2 is a protein that functions as the main switch for antioxidant defense in body cells [84]. This table also describes anti-inflammatory mechanisms and modulation of gene expression. Finally, it includes the role of polyphenols in mitochondrial function and autophagy.

Figure 1 shows an integrated molecular framework for modulation of oxidative stress mediated by how polyphenols influence the biology of oxidative stress through complementary pathways, direct redox chemistry (HAT/SET and chelation), and redox-sensitive signaling (Keap1–Nrf2/ARE). Similarly, mitochondrial-linked quality control is involved, while translation is limited by bioavailability and metabolism.

### 2.2. Dietary Sources of Polyphenols

The actual contribution of polyphenols depends largely on the type of food, the fraction consumed (pulp, skin, seeds, shell, etc.), the climatic and cultivation conditions of the input plant, the extraction and processing procedure, which together can enhance or decrease its concentration of bioactive compounds [31].

Vegetables, fruits, legumes, whole grains and certain beverages such as tea and red wine are rich in polyphenols, each with a variable composition of these bioactive compounds. Apples (*Malus domestica*) and grapes (*Vitis vinifera* L.) are well-studied sources. Each contains different varieties of polyphenols that can vary significantly depending on the cultivar and region of origin, influencing their antioxidant capacity and overall health benefits [95]. Similarly, other fruits such as strawberries and olives are also recognized for their polyphenol content, which can be modified by local climatic factors and agronomic practices [96,97].

The polyphenolic profile of plants is influenced not only by their genetic makeup (cultivar) but also by the geographical and climatic conditions of the place where they are grown [98]. Factors such as temperature, sun exposure, and soil characteristics play an important role in modulating the quantity and type of polyphenols produced in various plant species [6].

In this regard, it is interesting to note that citrus fruits from southern Spain, such as oranges and lemons, grown in the Mediterranean region, benefit from high sun exposure, which increases their flavonoid content and enhances their antioxidant capacity [99,100]. Furthermore, a study investigating the polyphenolic profiles of different apple cultivars in northeast China revealed that geographical location significantly affected their antioxidant activity and polyphenolic composition [101]. This highlights the importance of both cultivar selection and environmental conditions in agricultural practices. Similarly, research on the polyphenolic composition of olives in Tuscany, Italy, has shown that varietal differences, along with the unique agroclimatic conditions of the region, significantly affect the levels of beneficial health compounds, such as oleuropein [102]. Differences in soil composition and rainfall would also contribute to the variation in polyphenol concentrations in cultivated olives [103].

Table 4 describes some of the main food sources of polyphenols, including fruits and vegetables, beverages, oils and fats, spices, seasonings, legumes, and also seeds.

### 2.3. Flavonoids: Antioxidant Properties

The choice of flavonoids over other polyphenols is based on their status as a species of paramount scientific interest among polyphenols. In addition, they constitute the largest and best-characterized family of polyphenols, allowing the study of numerous structure-activity relationships within a single coherent chemical framework (C_6_–C_3_–C_6_) [9]. Flavonoids are present in all plant sources and derivatives. In addition, they are involved in numerous biological pathways with strong scientific evidence base and a large body of in vitro, in vivo, and clinical data, which helps reduce uncertainties [132]. Finally, thanks to their clear structure and functional groups, flavonoids are ideal for the development of QSARs, docking, molecular dynamics, nanoformulations, and delivery systems, as well as for testing novel technological or biotechnological platforms.

Flavonoids possess antioxidant properties that function through multiple mechanisms, including the direct scavenging of reactive oxygen species (ROS) [34,133]. The hydroxyl groups present in the aromatic structures of flavonoids allow them to neutralize free radicals and reduce oxidative stress, a pathological process associated with many chronic diseases [56,134]. These antioxidant capacities are attributed to the polyphenolic structure of flavonoids, which enables strong interactions with oxidative species through hydrogen bonds and other chemical interactions [135]. However, the antioxidant mechanisms of flavonoids extend beyond the simple elimination of free radicals. They have been shown to modulate cellular antioxidant defense systems while simultaneously improving the expression of endogenous antioxidant enzymes [134]. In addition, they also regulate biomarkers of oxidative stress and modulate signaling pathways involved in cellular redox homeostasis [136]. The broad antioxidant activity of flavonoids contributes significantly to protection against pathologies directly related to oxidative stress, such as cardiovascular disease, diabetes, and some neurodegenerative diseases [34].

Table 5 summarizes, in a comparative way, the main sources of bioactive plants rich in flavonoids (and some representative flavonoids), specifying the predominant compounds, the molecular mechanisms (antioxidant activity, anti-inflammatory activity, signal modulation, and genetic regulation) and the potential health benefits derived from these mechanisms.

### 2.4. Analytical and Classification Limitations and Evidence Quality Considerations

Table 6 shows some limitations and possible contradictions that summarize some of the evidence for the main mechanisms described in this section and highlights some sources of inconsistency.

## 3. Reduction in Oxidative Stress and Antioxidant Mechanisms

Flavonoids mitigate oxidative stress through multiple mechanisms, including direct ROS scavenging and modulation of cellular antioxidant defense systems [34]. The reduction in oxidative stress by flavonoids contributes substantially to their protective effects against endothelial dysfunction and cardiovascular disease [152,153].

### 3.1. Fundamental Mechanisms and Implications for Health

Oxidative stress represents a critical pathological condition that reflects impaired cellular redox homeostasis and occurs when ROS generation exceeds the capacity of antioxidant and detoxification systems to neutralize reactive intermediates [12]. More formally, oxidative stress is defined as an imbalance between oxidants and antioxidants, which ultimately favors oxidants, leading to impaired redox signaling and control, or molecular damage [154,155]. ROS are physiologically generated during normal metabolism, particularly through mitochondrial electron transport and enzymatic sources (e.g., NADPH oxidases, xanthine oxidase), as well as non-enzymatic reactions [133,156,157].

The consequences of oxidative stress extend beyond direct molecular damage. When ROS levels exceed cell antioxidant and repair capacity, oxidative stress can promote lipid peroxidation, protein oxidation, and DNA damage [158,159]. These molecular alterations can compromise cellular integrity and function and are involved in the development and progression of multiple chronic diseases [12,14,15]. The relationship between oxidative stress and disease development is particularly evident in cardiovascular diseases, diabetes, neurodegenerative disorders, and cancer, where oxidative stress acts as a common pathogenic mechanism [13].

### 3.2. Oxidative Stress in the Development of Chronic Diseases

The role of oxidative stress in the pathogenesis of chronic diseases has been extensively documented in multiple conditions [16]. In cardiovascular diseases, such as coronary artery disease, myocardial infarction, and heart failure, oxidative stress contributes to endothelial dysfunction, vascular inflammation, and myocardial injury [160]. An imbalance between ROS production and antioxidant defense capacity can amplify inflammatory signaling and promote apoptosis, thus accelerating disease progression in susceptible tissues [161]. Similarly, in neurodegenerative disorders, oxidative stress is involved in neuronal dysfunction and cell loss through mechanisms including lipid peroxidation, protein oxidation, mitochondrial impairment, and dysregulated redox signaling during pathological insults [162,163].

The development of some cancers is also closely linked to oxidative stress, which refers to a state in which the production of oxidants exceeds antioxidant defenses, leading to disruption of redox signaling and/or molecular damage [12,13,14]. The imbalance of oxidative stress acts as a trigger for malignant mutations, setting off a sequence of immunosuppressive mechanisms and damaging cellular responses [164]. In metabolic disorders, particularly diabetes mellitus, oxidative stress appears to be a particularly concerning factor, with metabolic oxidation being a major contributor to insulin-dependent and non-insulin-dependent diabetes [165].

### 3.3. Antioxidant Mechanisms of Bioactive Compounds Derived from Plants

#### Elimination of Free Radicals and Neutralization of ROS

Some plant-derived polyphenols can exert antioxidant effects through direct radical scavenging, in which phenolic hydroxyl groups donate an electron or a hydrogen atom to stabilize reactive radicals, thus limiting propagating reactions such as lipid peroxidation [56,166]. A good example is gallic acid, which has been widely used as a reference phenolic antioxidant in chemical and cellular systems due to its measurable ability to scavenge such radicals [167]. Similarly, polyphenols with multiple phenolic groups, including tannic acid, a typical plant polyphenol, can inhibit reactive species in model systems and reduce oxidative damage under defined experimental conditions [168]. It is important to note that the direct scavenging capacity varies considerably between different compounds and depends on structural characteristics (such as the number and position of hydroxyl groups), the physicochemical environment and the specific radical/oxidant being evaluated [157].

Furthermore, although radical scavenging activity is readily demonstrated in vitro, its quantitative contribution in vivo can be similarly limited by bioavailability, conjugation, and tissue exposure [2]. These factors often shift dominant biological effects toward modulation of endogenous antioxidant defenses, rather than stoichiometric neutralization of ROS [169]. Consequently, flavonoids, resveratrol, and catechins have demonstrated ROS-reducing effects in multiple in vitro and in vivo models. Translating these findings into therapeutic outcomes requires careful consideration of dose realism, metabolite activity, and clinically relevant endpoints [49].

### 3.4. Improvement of Endogenous Antioxidant Defense Systems

In addition to direct ROS scavenging, plant-derived polyphenols can contribute to cytoprotection by improving endogenous antioxidant defense systems [170]. Glutathione (GSH) is a central intracellular antioxidant involved in peroxide detoxification and maintenance of redox homeostasis, including through glutathione-dependent enzymes, and can mitigate oxidative and nitrosative stress under defined conditions [171]. Some research suggests that plant-derived compounds and extracts (such as *Taraxacum officinale*) modulate components of the glutathione system, which could enhance cellular antioxidant capacity in experimental models [172,173].

Endogenous antioxidant defenses also include enzymatic components such as SOD, catalase, and GPx, which coordinate ROS detoxification [174]. Importantly, modulation of these systems is frequently associated with oxidation–reduction-sensitive transcriptional programs, as in the case of Nrf2/ARE [29]. This provides a mechanistic basis for the sustained upregulation of cytoprotective enzymes beyond the immediate stoichiometric clearance capacity of the compounds themselves. Table 7 summarizes representative plant bioactives and their associated enzymatic defense responses, as reported in various medicinal species [15].

### 3.5. Mechanistic Evidence Appraisal: Limitations, Contradictions, and Strength of Evidence

To strengthen the critical evaluation of the evidence presented in this section, Table 8 summarizes the most important limitations observed and some sources of contradiction in the different types of studies.

## 4. Polyphenols for Disease Prevention and Therapeutic Applications Flavonoid Derivatives

Among the main documented activities of flavonoids are antioxidant, anti-inflammatory, and anticancer effects, mainly attributed to their unique chemical structures, particularly their hydroxyl groups [9,117].

Some specific flavonoids are responsible for inducing diverse bioactivities. Glycosylated flavonoids often improve solubility, increasing bioavailability [175]. Rutin, derived from various plant sources, has been recognized for its antitrypanosomal effects, implying its potential in the treatment of infectious diseases [176]. Furthermore, some flavonoids, such as nobiletin, have been associated with metabolic health, offering protective effects against conditions such as non-alcoholic fatty liver disease [177]. This means that the diverse bioactivities of flavonoid derivatives are supported by scientific evidence and have great potential in nutraceutical and pharmaceutical applications.

Plant-derived bioactives have been investigated for their preventive and therapeutic relevance, based on evidence from preclinical models and, where available, human studies [178,179]. Particular emphasis is placed on cardiovascular diseases, given their significant contribution to overall mortality. Recent studies have identified structurally diverse flavonoid derivatives with enzyme-targeting activities [180]. However, the degree to which these mechanisms transcend experimental systems remains variable and context-dependent [181].

### 4.1. Prevention and Treatment of Cardiovascular Diseases

Cardiovascular diseases, such as coronary artery disease, myocardial infarction, and heart failure, are the leading causes of mortality and disability worldwide [160]. Oxidative stress plays a key role in cardiovascular pathogenesis, with increased ROS production in the myocardium leading to heart failure with preserved ejection fraction (HFpEF) [182]. The antioxidant and anti-inflammatory properties of flavonoids mitigate oxidative stress, a key factor in endothelial dysfunction and hypertension [9,183,184,185]. These bioactive compounds improve endothelial function by promoting the bioavailability of NO (nitric oxide), a gaseous signaling molecule primarily produced by the vascular endothelium. Its central function is to induce vasodilation by relaxing smooth muscle and to maintain vascular homeostasis [186]. Modulation of vasoactive factors involved in blood pressure regulation, such as angiotensin-converting enzyme activity, represents an additional mechanism through which flavonoids can improve endothelial function [187].

#### 4.1.1. Some Molecular Pathways and Signaling Mechanisms Associated with the Vascular Effects of Flavonoids

Research has shown that flavonoids modulate the PI3K-AKT pathway, improving endothelial function and vasodilation, while reducing oxidative stress, thus promoting cardiovascular health [188]. Specifically, anthocyanins can improve endothelial function by modulating the endothelial nitric oxide synthase (AKT) pathway and transcription factors [189]. In this regard, flavonoid consumption can improve endothelial function by activating adenosine monophosphate-dependent protein kinase (AMPK) and related signaling cascades [190]. Some studies have shown that homoplantaginin, a flavonoid extracted from plants with traditional medicinal use, especially in the genera *Salvia* and *Plantago*, attenuates high-glucose-induced apoptosis of vascular endothelial cells by promoting autophagy through the AMPK/TFEB pathway [94]. These molecular mechanisms highlight the multifaceted ability of flavonoids to restore and maintain endothelial function through various signaling pathways.

Table 9 summarizes the main modulated signaling pathways associated with the vascular effects of flavonoids and their relationship to vascular and metabolic function. The PI3K-AKT pathway, AMPK signaling, and NO bioavailability are highlighted, with a description of the biological function of each pathway, the mechanistic effect of flavonoids, and the most representative compounds. This synthesis allows an integrated view of how different flavonoids converge in key pathways involved in endothelial protection, energy homeostasis, and blood pressure regulation.

#### 4.1.2. Prevention and Management of Cardiovascular Diseases. Reduction in Cardiovascular Risk

Resveratrol, which is abundant on the skin of red grapes and in *Polygonum cuspidatum*, has shown wide benefits for human health [195]. This compound is notable for its positive effects on cardiovascular health, as it is linked to its ability to scavenge reactive oxygen species (ROS) and exhibit antioxidant and anti-inflammatory properties [196]. Some research has shown that resveratrol is able to restore hypertension-induced liver damage in rats, while the hepatic antioxidant defense system contributes to the systemic redox status [197]. An excessive increase in ROS and/or a decrease in antioxidant defense systems triggers oxidative stress, which plays an influential role in the pathogenesis of hypertension and organ damage related to hypertension [198]. Other clinical studies have shown that resveratrol supplementation decreases systolic and diastolic blood pressure [199]. The cardiovascular benefits of polyphenols extend beyond the reduction in blood pressure and include improvements in lipid profiles and endothelial function [90,125,200,201]. Quercetin, a flavonol, exhibits cardiovascular benefits such as lowering blood pressure, reducing cholesterol levels, and improving vascular function [202]. These cardiovascular benefits are mediated by multiple mechanisms, including reducing oxidative stress, inhibiting inflammatory pathways, and improving endothelial function [203].

Numerous studies have shown several advantages associated with the Mediterranean diet. In terms of health, it is associated with a better cardiometabolic profile and a lower risk of cardiovascular events, largely due to its high content of fiber, polyphenol, and monounsaturated fat [204]. This diet is rich in polyphenol-containing foods, such as extra virgin olive oil (EVOO), which has a strong impact on protection against cardiovascular disease [200,205,206]. In this regard, hydroxytyrosol and tyrosol, the main phenolic compounds in olive oil, show promise in cardioprotection by combating oxidative stress, improving lipid profiles, and modulating inflammation [201]. Tyrosol attenuates lipopolysaccharide-induced inflammation in human umbilical vein endothelial cells (HUVECs), promoting vascular health against atherosclerosis [207]. Some research estimates that replacing conventional refined olive oil with EVOO in phenols would produce a cholesterol-reducing effect, not related to fatty acid content, suggesting that active polyphenolic compounds in EVOO have beneficial effects on cardiovascular health regardless of the basic lipid profile [125].

However, it is important to interpret the evidence surrounding Mediterranean dietary patterns with caution. This is because other observational studies may have been influenced by biased interpretive factors, such as socioeconomic status, certain health habits, and inaccuracies in self-reporting methods of food [208]. Furthermore, while randomized controlled trials can provide stronger evidence on Mediterranean dietary interventions, they also have limitations, particularly in terms of adherence and generalizability [209]. Specifically, claims linking Mediterranean diet patterns to the antioxidant effects of polyphenols are not conclusive and should be considered correlational rather than causal [210].

#### 4.1.3. Endothelial Protection and Vascular Function

The protective effects of flavonoids against cardiovascular disease extend to multiple vascular beds and pathological states, including atherosclerosis, hypertension, and myocardial infarction [184]. These protective effects are mediated by improvements in endothelial function, reduction in oxidative stress, and mitigation of inflammatory processes [152,185,191,211]. These protective mechanisms are particularly important in the context of atherosclerosis, where endothelial dysfunction and vascular inflammation represent critical pathological processes [212].

Consumption of flavonoid-rich foods has also been associated with improved endothelial function, measured by flow-mediated dilation (FMD), indicating NO-mediated endothelial vasodilation [213]. Cocoa and chocolate, which are rich in flavonoids and proanthocyanidins, reduce blood pressure levels and cardiovascular risk, with improvements in measures of vascular health, including arterial stiffness and endothelial function, possibly due to increased NO production and antioxidant/anti-inflammatory properties [214].

Table 10 describes how flavonoids contribute to cardiovascular protection from various angles. Their effects on blood pressure regulation (modulation of vasoactive factors, increased nitric oxide levels, and improved endothelial function) are evident. In addition, their role in specific conditions such as atherosclerosis, hypertension, and vascular inflammation is highlighted, highlighting their antioxidant and anti-inflammatory mechanisms. Finally, the concept of endothelial dysfunction is integrated as a central pathogenic axis, demonstrating how the action of flavonoids on oxidative stress, cytokines, and adhesion molecules contributes to maintaining vascular health.

### 4.2. Prevention of Metabolic Diseases and Management of Diabetes

Metabolic disorders, particularly in the case of insulin-dependent and non-insulin-dependent diabetes mellitus, are characterized by oxidative stress as a central pathogenic mechanism [165,232].

Some research shows that phenolic compounds reduce the risk of metabolic syndrome and type II diabetes [72]. Specific plant bioactives, such as components of ginger essential oil, have demonstrated antioxidant activities that can contribute to metabolic health [233]. Extra virgin olive oil has also been shown to enhance antidiabetic and antihyperlipidemic activities by modulating oxidative stress and lipid metabolism [234]. This improves glycemic control while reducing cardiovascular risk factors in diabetic patients [235,236]. Furthermore, resveratrol inhibits human salivary and pancreatic α-amylase, which may contribute to better glycemic control [67].

### 4.3. Prevention and Treatment of Neurodegenerative Diseases

Neurodegenerative diseases, such as Alzheimer’s, Parkinson’s, and amyotrophic lateral sclerosis, are characterized by progressive neuronal loss and dysfunction [196]. Oxidative stress plays an important role in the pathogenesis of these conditions, as ROS accumulation causes neuronal damage and even cell death [163].

Uric acid, which functions as an endogenous antioxidant, scavenges free radicals and reactive oxygen species (ROS), thus alleviating oxidative stress and preventing neuronal damage [237]. Plant-derived compounds that enhance similar antioxidant mechanisms may provide neuroprotective effects. Cannabidiol (CBD), a plant-derived compound, has demonstrated the ability to reduce ROS production while simultaneously improving mitochondrial function, which is crucial for cellular health in the context of neurodegenerative diseases [162]. In this regard, polyphenols cross the blood–brain barrier and are used in the treatment of various neurodegenerative diseases, including Alzheimer’s disease, Parkinson’s disease, Huntington’s disease, and amyotrophic lateral sclerosis (ALS) [238]. By mitigating oxidative damage, CBD can slow the progression of neuronal death, offering hope for conditions such as Alzheimer’s disease, where oxidative stress and inflammation are central pathogenic mechanisms [239].

Members of the microRNA-181 family have been identified as small, non-coding RNA molecules that act as regulators of gene expression in the human body. By binding to messenger RNA (mRNA), they prevent the production of certain proteins. In this way, they play an important role in stroke pathogenesis, where oxidative stress contributes to cellular and molecular damage during post-ischemic trauma [163]. Therefore, plant-derived bioactive compounds that modulate oxidative stress responses could have therapeutic potential to prevent secondary brain damage after stroke, as well as other neurodegenerative diseases.

Hydroxytyrosol, for its part, is responsible for the destabilization of α-synuclein oligomers, which are implicated in the pathogenesis of Parkinson’s disease [240]. The amphipathic nature of hydroxytyrosol allows it to interact with aqueous and lipid environments, facilitating its interaction with α-synuclein oligomers [85]. Resveratrol, however, shows therapeutic potential in amyotrophic lateral sclerosis [141], demonstrating potential benefits in vascular cognitive impairment [66]. Here, as in the case of cardiovascular disease, a Mediterranean diet, rich in foods containing polyphenols, has been associated with a lower incidence of neurodegenerative diseases and improved cognitive function [241,242].

### 4.4. Cancer Prevention and Therapeutic Potential

The development of some cancers is closely related to oxidative stress, and its imbalance acts as a trigger for mutations with malignant characteristics [164]. Polyphenols can exhibit anticancer effects through multiple mechanisms, including induction of apoptosis, inhibition of cell proliferation, and modulation of oncogenic signaling pathways [243,244]. Furthermore, cyanidin-3-O-glucoside (C3G), the predominant anthocyanin in berries, exhibits antioxidant and anticancer properties with potential therapeutic benefits for gastric cancer [52]. In some cases, high intake of phenolic compounds has been associated with a lower risk of certain cancers [72].

In this regard, foods such as red wine, rich in flavonols and anthocyanins, provide multiple anticancer compounds [48]. Extra virgin olive oil (EVOO) and its phenolic extracts also demonstrate potential anticancer effects in hepatocellular carcinoma cells [245]. Evaluation of EVOO phenols on cytotoxicity in cells of hepatocellular carcinoma has shown effects on liver cancer [246]. Curcumin and resveratrol, for their part, have demonstrated cytotoxic effects against lung cancer cells [247]. The combination of these two polyphenols could provide greater anticancer efficacy compared to traditional monotherapy [43]. However, this opinion is not shared by all researchers.

Plant-derived compounds that modulate ROS levels may also offer therapeutic potential. Salicylic acid (SA), a plant-derived antioxidant, can paradoxically exert a pro-oxidant effect in vitro and exhibit an antitumor effect in vivo by reactivating intercellular ROS signaling [248]. This dual mechanism demonstrates the complexity of antioxidant action in cancer therapy, where a controlled increase in ROS can enhance therapeutic efficacy.

### 4.5. Inflammatory and Autoimmune Diseases

Inflammatory and autoimmune diseases represent a significant burden on global health, characterized by dysregulated immune responses that lead to chronic inflammation and progressive tissue damage [249]. Rheumatoid arthritis (RA) is a prime example of this health problem. It is a typical autoimmune disorder that exemplifies this pathological process through persistent synovial inflammation, progressive joint destruction, and systemic complications [250]. Among dietary interventions, polyphenolic compounds have attracted considerable scientific attention. Phenolic compounds show promising potential in the prevention and treatment of such diseases by modulating inflammatory pathways [251]. Furthermore, polyphenols in extra virgin olive oil exert anti-inflammatory effects on microglial cells through the TREM2 signaling pathway [89]. This mechanism is particularly relevant in neuroinflammatory and neurodegenerative diseases.

Flavonoids also exert anti-inflammatory effects through modulation of the NF-κB and NLRP3 inflammasomes, critical intracellular signaling complexes involved in the pathogenesis of numerous metabolic and inflammatory diseases [252,253]. Molecular assays show that catechin, apigenin, and epicatechin exhibit a high binding affinity to NLRP3 PYD, comparable to the NLRP3 inhibitor MCC950 [254]. These findings indicate that flavonoids can directly interact with and inhibit inflammasome activation, thus suppressing pro-inflammatory cytokine production and simultaneously mitigating chronic inflammation and its sequelae in various diseases [255].

Flavonoids also inhibit the regulatory activity of certain enzymes and transcription factors involved in inflammation [256]. Specifically, studies show that flavonoids can inhibit cyclooxygenase (COX) enzymes, which are central mediators of inflammatory responses [257]. The polyphenolic structure of flavonoids allows for strong interactions with COX-2 active sites through hydrogen bonds and hydrophobic forces, enabling them to function as natural COX-2 inhibitors [135].

The nuclear factor kappa B (NF-κB) pathway also plays an important role in inflammatory activity, and the inhibition of NF-κB-mediated inflammation represents a potential strategy for the treatment of inflammatory diseases [252]. In this regard, flavonoids can modulate NF-κB activation, thus reducing the expression of inflammatory genes and the production of pro-inflammatory mediators [228]. In endothelial cells, anti-inflammatory activity has been attributed to modulation of NF-κB activation [258]. Furthermore, some research has shown that flavonoids inhibit the activation of the hepatic TLR4/MyD88/NF-κB pathway, effectively improving liver injury and reducing systemic inflammation [259].

Among the more specific polyphenolic compounds and their clinical anti-inflammatory effects is curcumin, the main active polyphenol in turmeric. Resveratrol, a stilbene polyphenol found in grapes, berries and red wine, has demonstrated antioxidant and anti-inflammatory effects in models of rheumatoid arthritis (RA). Quercetin, a flavonoid abundant in apples, onions, and tea, exhibits interesting anti-inflammatory properties. Theaflavins are the main polyphenols in black tea. Extra virgin olive oil (EVOO), a cornerstone of the Mediterranean diet, also contains abundant polyphenols, such as oleuropein, hydroxytyrosol and tyrosol, which can exert antioxidant and anti-inflammatory effects on peripheral blood mononuclear cells in patients with RA.

### 4.6. Limitations and Contradictions

The evidence summarized in this section is heterogeneous and often depends on the context in which it is presented. The main limitations and contradictions between different areas of the disease are summarized in Table 11.

## 5. Bioavailability, Clinical Efficacy and Encapsulation of Polyphenols (Flavonoids)

The therapeutic efficacy of bioactive substances of plant origin depends primarily on their bioavailability and pharmacokinetic properties [260]. The degree to which these compounds are absorbed, distributed, and reach target tissues is influenced by multiple factors, such as chemical structure, food matrix, intestinal microbiota composition, and individual genetic factors [261]. In other words, there is a high dependence not only on their in vitro antioxidant capacity but also on their absorption, distribution, and bioavailability in vivo [12]. In this regard, it is observed that resveratrol, for example, undergoes transformation through multiple mechanisms that could affect its stability and bioactivity [262]. However, quercetin, a well-studied flavonoid widely present in fruits, vegetables, and tea, shows that its bioavailability is significantly improved in nanoformulations [263].

In general, recent clinical evidence suggests potential benefits of flavonoid-rich interventions on cognitive/neuroprotective outcomes, modulation of cardiometabolic risk, and inflammatory/immunometabolic endpoints, while heterogeneity in populations, dosage, matrices, and outcome selection limits comparability [264,265,266,267,268,269,270,271,272,273,274,275,276,277].

### 5.1. Bioavailability and Metabolism of Polyphenols (Flavonoids)

The bioavailability of flavonoids is a fundamental element to consider as a result of the number of internal and external variables to which a particular compound can be subject. Understanding these factors of change is crucial, as their beneficial health effects are profoundly influenced by their absorption, distribution, metabolism, and excretion in the human body [278,279]. Therefore, variables such as the food matrix, enzyme digestion, and microbial fermentation could significantly affect the bioavailability of these compounds [280,281,282,283]. Bioavailability can also be affected by dietary interactions, such as the presence of certain types of fats, which could facilitate the absorption of fat-soluble flavonoids [279].

The complex interplay between food structures, digestive physiology, and microbial activity suggests that optimizing flavonoid bioavailability requires a multifaceted approach that considers these diverse influences to ensure success.

Some clinical evidence suggests potential benefits of flavonoid-rich interventions on cognitive/neuroprotective outcomes [284]. They also modulate cardiometabolic risk and inflammatory/immunometabolic endpoints, while heterogeneity in populations, doses, matrices, and outcome selection limits comparability [285].

### 5.2. Factors That Can Affect Bioavailability

#### 5.2.1. Chemical Structure and Food Matrix

The chemical structure of flavonoids can significantly affect their solubility, stability, and absorption [286]. The presence of glycosylated forms, such as sugars bound to flavonoid structures, can influence their digestive stability and, therefore, their bioaccessibility [287]. Flavonoid glycosides must undergo hydrolysis to release the aglycone form and therefore exhibit their biological activity [286]. Free flavonoids, typically in the form of aglycones, have a higher absorption rate compared to their bound counterparts, which adhere to cell wall materials [288]. This characteristic suggests that methods that improve aglycone release, such as fermentation, can significantly increase their bioavailability [289]. Strategies to improve flavonoid bioavailability often involve chemical modifications such as glycosylation, which can improve solubility and stability [290]. These glycosylation modifications could influence their solubility, absorption, and overall bioavailability. Glycosylation tends to improve the stability of flavonoids against enzymatic degradation, increasing their water solubility. This promotes better absorption in the gastrointestinal tract [291]. However, complex structures and conjugated forms of flavonoids in plant matrices can sometimes hinder bioavailability by reducing their release during digestion [292].

Some flavonoids, particularly flavanones, exhibit varying degrees of bioaccessibility, mediated by factors such as the food matrix and the processing methods used during food preparation [293]. The food matrix can enhance or hinder the release of flavonoids during digestion. Some research indicates that flavonoids present in complex matrices (such as dark chocolate or some vegetables) are better protected during gastrointestinal digestion, allowing for better preservation and possibly better absorption [294].

#### 5.2.2. Bioaccessibility, Digestion, and Metabolism

Bioaccessibility is defined as the portion of a compound that is released from the food matrix and becomes available for absorption during digestion, making it a determining factor of bioavailability [295]. The stability of flavonoids during in vitro digestion processes plays a fundamental role in their subsequent absorption [296]. Some flavonoids can undergo extensive degradation during gastric and intestinal digestion, negatively impacting their net final bioavailability [297,298]. Similarly, the interaction between flavonoids and digestive enzymes can also affect their bioavailability. Enzymes such as α-amylase may modulate flavonoid absorption in the intestine, thus influencing overall metabolic responses [299].

The digestive process may also influence the bioavailability of flavonoids, as they undergo biotransformation and metabolism in the gastrointestinal tract. Following ingestion, various flavonoids can be hydrolyzed by bacterial enzymes in the intestinal tract, releasing their aglycone forms [283]. This process is crucial, as the bioactivity of flavonoids often depends on their transformation by the gut microbiota, resulting in bioactive metabolites that can exert beneficial effects in the human body [300]. Hesperidin, for example, is a flavonoid found in citrus fruits that exhibits greater anti-inflammatory activity in its aglycone form than in its glycosylated form [301].

#### 5.2.3. Particle Size and Extraction Preparation

Some research has highlighted the impact of particle size on flavonoid bioavailability. Obtaining smaller particles (nanoparticles) through processing techniques such as microfluidization or ultrasonication has demonstrated improved bioavailability [302]. This is due to a larger surface area for absorption during digestion and interaction with intestinal membranes. Extracts derived from optimized plant materials for particle size could significantly improve the release and absorption of flavonoids during digestion [303].

Regarding the preparation of extracts, fermentation processes can improve the bioavailability of flavonoids [304]. Using lactic acid bacteria in solid-state fermentations, bound forms of flavonoids can be hydrolyzed, resulting in increased flavonoids release with improved bioavailability [305]. This mechanism is consistent with findings suggesting that fermented foods, such as yogurt or kimchi, can provide higher concentrations of bioactive compounds, leading to more significant health benefits [8,306].

#### 5.2.4. Role of Metabolites and the Intestinal Microbiota

Flavonoids can modulate the composition and activity of the gut microbiota, which can subsequently influence overall health. Polyphenols, including flavonoids, act as prebiotics, promoting the growth of beneficial gut bacteria and inhibiting the harmful microbiota [307]. This modulation of the gut microbiota is essential for optimizing nutrient absorption and thus maintaining gut health, contributing to the general health benefits of flavonoids [308].

It is important to note that after flavonoid ingestion, metabolic conversion occurs primarily in the intestines and liver [283]. The formation of various metabolites could enhance or even inhibit their bioactive properties. Some metabolites exhibit greater bioactivity than the parent compounds, which could contribute to the health effects associated with flavonoids [181]. Furthermore, the gut microbiota participates in the fermentation and metabolism of flavonoids, which could lead to the production of bioactive metabolites that may offer health benefits [293,309]. Metabolic byproducts of flavonoid interactions within the gut microbiome may also possess bioactive properties that further enhance the biological effects of flavonoids, providing multifaceted health benefits [310].

#### 5.2.5. Implications for Health and Nutrition

Improved flavonoid bioavailability could result in increased antioxidant capacity, anti-inflammatory effects, and potential protective benefits against chronic diseases such as cardiovascular disease, cancer, and diabetes [293,295,311]. Therefore, dietary strategies that optimize the bioavailability of these compounds are essential. This may include consuming specific food combinations, using processing techniques that promote the stability of flavonoids, and potentially, as previously mentioned, using encapsulation methods to improve gastrointestinal absorption [312].

The differential bioavailability of flavonoids and their metabolites would have substantial implications for human health. The presence of flavonoids in the diet can modulate various physiological pathways, such as arterial dilation and inflammation reduction, which are crucial to maintaining cardiovascular health [290].

In addition, various approaches are being developed to improve the bioavailability of flavonoids. Micronutrient combinations or targeted delivery systems using nanotechnology represent one approach to improving the efficacy of flavonoids as therapeutic agents [313]. In this case, co-administration of flavonoids with micronutrients such as zinc or vitamin C can enhance their antioxidant activity and, therefore, have better therapeutic effects in some chronic diseases [314].

### 5.3. Interactions Between Polyphenols (Food Matrix, Microbiome, Drugs/Nutrients)

Analyzing the interactions between polyphenols, the food matrix, the gut microbiome, and concomitant drugs is crucial to understanding their function and potential efficacy or contraindication. Food components can improve or inhibit the absorption of polyphenols, which could obviously lead to variations in their beneficial health effects [315]. The synergistic effects of polyphenols when consumed with certain dietary fibers and probiotics have demonstrated increased bioaccessibility and biological effects mediated by fermentation of the microbiome [316].

Drug–food interactions are another factor of great importance in clinical practice, especially in the metabolism with different drugs [317]. Since the microbiome plays a fundamental role in the metabolism of polyphenols to bioactive forms, understanding these interactions is key to formulating more effective nutraceuticals [284,318,319,320,321]. However, it is important to consider that these interactions can lead to changes in the efficacy or toxicity of medications. Therefore, physicians must take into account the’ dietary habits of patients when prescribing different drugs, as the risk of adverse drug reactions increases significantly in these situations [322].

Foods rich in polyphenols can modulate the activity of cytochrome P450 enzymes, which could alter the pharmacokinetics of co-administered drugs [323,324]. Modulation of cytochrome P450 enzymes is also critical in polyphenol metabolism and can be significantly influenced by diet choices [325,326]. Certain flavonoids have been found to inhibit drug-metabolizing enzymes, affecting their pharmacokinetics. Cytochrome 3A4 also metabolizes some commonly prescribed drugs [327].

Flavonoids can also interact synergistically with drugs to enhance therapeutic efficacy and reduce adverse effects. The pharmacokinetics of many drugs can be significantly altered by the presence of dietary flavonoids, which can influence drug metabolism and bioavailability [328]. Flavonoids can inhibit specific drug-metabolizing enzymes, thus improving their bioactivity and reducing the need for higher doses [329].

Incorporation of flavonoid-rich extracts into pharmaceutical formulations could even improve drug stability and efficacy, as observed in interactions with human serum albumin [330]. This interaction would improve the bioavailability of various compounds by facilitating their transport in the bloodstream [331]. Understanding how dietary flavonoids interact with these metabolic pathways is essential to avoid possible undesirable drug–nutrient interactions and thus ensure optimal therapeutic efficacy.

### 5.4. Interactions with Other Compounds (Dietary Components, Drugs, Supplements) Some Related Mechanisms: Chelation, Antioxidant Activity, Synergies, and Microbiota

#### 5.4.1. Chelation of Metal Ions

Flavonoids possess unique structural attributes that allow them to chelate metal ions [332]. This property is crucial not only for heavy metal detoxification, but also for facilitating their bioavailability in biological systems [333]. Some studies highlight the chelating capacity of flavonoids, which is often derived from their functional hydroxyl and carbonyl groups [334]. These groups bind to transition metals such as iron and copper, thus mitigating the oxidative stress resulting from free radical reactions catalyzed by these metals [335]. Some research has shown that quercetin competes effectively with metals such as cadmium, thereby reducing their harmful effects on cellular systems [336,337]. It is also important to highlight the role of dietary polyphenols in regulating iron homeostasis and supporting gut microbiota health through chelation mechanisms [307]. This chelation not only improves the effectiveness of flavonoids as antioxidants but also helps mitigate the bioavailability of toxic metals in the human body [87].

#### 5.4.2. Biomolecular Interaction of Flavonoids

The function of flavonoids extends to various biological systems, where they interact with crucial biomolecules such as proteins and DNA, facilitating protective effects against oxidative damage [181]. The protective function of nobiletin against arsenic-induced liver damage underscores its potential as a therapeutic agent through antioxidant mechanisms [338]. Furthermore, kaempferol has shown promise in counteracting cancer cell proliferation due to its antioxidant activity, further supporting the utility of flavonoids in clinical applications [309].

Some evidence suggests that dietary flavonoids can modulate the composition of the gut microbiota and, conversely, that metabolites derived from the microbiota can contribute significantly to systemic effects [283]. This bidirectional relationship introduces interindividual variability in exposure and response, which must be considered when translating mechanistic findings into human health outcomes [307,308,310].

### 5.5. Microbiotransformation Driven by the Microbiota and Interindividual Variability

Bioaccessibility (the fraction released from the food matrix during digestion) is an important factor in absorption and varies with processing, co-ingested macronutrients, and the physicochemical properties of flavonoids [4]. Following absorption, first-pass metabolism in the enterocyte and liver can substantially reduce circulating concentrations of the parent compound, while generating conjugated metabolites with differentiated distribution and activity [300].

### 5.6. Some Clinical Evidence Related to Dietary Flavonoids

Concomitant consumption of food, nutraceutical ingredients, and medications can influence the absorption and metabolism of flavonoids through shared transporters and biotransformation pathways, potentially altering the efficacy or safety [323]. These interactions risks are particularly relevant in the case of high-dose supplements and polypharmacy, and should be monitored in clinical practice [322].

Current clinical evidence clarifies the efficacy and safety of polyphenols and flavonoids in various health outcomes [320,321,339,340,341,342]. Epidemiological studies and meta-analyzes indicate positive effects of long-term consumption of a diet rich in plant polyphenols, considering chronic conditions such as cardiovascular disease and type 2 diabetes [343]. This occurs primarily through mechanisms that modulate the responses of inflammation and oxidative stress, and even neurodegenerative disorders. Some research has found correlations between high dietary flavonoid intake and improved metabolic profiles [342]. However, evidence indicates that the health benefits derived from polyphenol consumption are dose-dependent and influenced by factors such as age, health status, and genetic predisposition [344].

It is important to note that, while substantial evidence supports the health benefits of polyphenols, discrepancies persist due to variations in study designs and populations. Therefore, integrating clinical evidence into diet guidelines and healthcare interventions remains a critical goal. The variability in individual responses to flavonoid intake underscores the need for personalized approaches to dietary recommendations [181]. Although several studies are very promising, more high-quality clinical trials are needed to establish optimal dosages and definitively assess the long-term health effects of flavonoid consumption [345,346].

Table 12 provides a concise synthesis of the representative clinical evidence and highlights the main design-related limitations that should guide interpretation.

### 5.7. Microencapsulation and Spray Drying

Another critical element is the preservation of polyphenol bioactivity. This presents a significant challenge, as these compounds are often highly susceptible to degradation by oxidation, hydrolysis, and thermal decomposition [347]. For this reason, encapsulation technologies have been developed to protect plant bioactives not only from external degradation, but also during digestion.

The encapsulation of flavonoids in delivery systems such as liposomes or nanoparticles could improve both their stability and bioaccessibility, potentially enhancing their therapeutic effects [314,348,349,350,351]. This encapsulation process should not only protect polyphenols from premature degradation but also allow for a more controlled release, thus improving their desired therapeutic effects. Similarly, encapsulated polyphenols have shown improved stability and absorption, suggesting enhanced pharmacokinetic profiles when integrated into delivery systems [352].

The effects of encapsulation and in vitro digestion on the anthocyanin composition and antioxidant activity of plant extracts have been systematically evaluated, demonstrating that processing can significantly affect the therapeutic efficacy of plant-derived compounds [167].

Among the various studies on encapsulation techniques, those using maltodextrin have been analyzed to preserve the activity of oleuropein-rich olive leaf extracts, with concentration assessments performed before and after encapsulation [353]. Microencapsulation approaches, including lyophilization, improve the stability and bioavailability of polyphenol extracts [354]. In this regard, lyophilization represents an effective stabilization method that preserves the bioactivity of polyphenols and allows long-term storage [355]. This technique removes water by sublimation at low temperature and pressure, minimizing the thermal degradation of heat-sensitive compounds [356].

### 5.8. Contributions, Limitations, and Contradictions

Table 13 shows a summary of the main contributions by area and the limitations and contradictions of this section.

## 6. Nutraceuticals and Regulatory Considerations

The nutraceutical industry, which represents the convergence of the nutritional and pharmaceutical industries, has experienced significant growth in recent years. This has been driven by the gradual increase in the awareness of consumers of the health benefits associated with dietary supplements [357,358].

To transform flavonoids into effective nutraceutical products, it is necessary to address the formulation of these compounds, their bioavailability, and the regulatory considerations surrounding their use. Formulation strategies that improve the bioavailability and stability of polyphenols in general and flavonoids in particular are crucial to their successful application in health products [359,360,361]. These formulations not only protect the active compounds from degradation, but can also enhance their penetration into different tissues, providing therapeutic benefits at lower or controlled doses [362,363].

The conversion of flavonoids into nutraceutical products requires careful consideration of both the formulation and the accompanying regulatory aspects. In this regard, various delivery methods have been explored, such as nanocrystals, liposomes, and cyclodextrin complexes, which aim to improve the bioavailability and efficacy of flavonoids when used as functional ingredients in food products or as dietary supplements [350,351]. Nanocarrier systems could improve the solubility and stability of flavonoids, allowing more effective applications in therapeutic nutraceuticals [314,364].

Furthermore, the regulatory framework plays a vital role in the approval, acceptance, and marketing of flavonoid-based nutraceuticals. Agencies such as the Food and Drug Administration (FDA) and the European Food Safety Authority (EFSA) have strict regulations governing the research, development, and marketing of flavonoids, focusing on their short, medium, and long-term safety and efficacy [365]. It is essential that flavonoid research adheres to the respective regulatory frameworks to ensure that medical claims about different types of flavonoids are supported by robust clinical evidence [359,366].

The marketing of flavonoid-based nutraceuticals is also governed by regulations from agencies such as the FDA and EFSA. However, distinctions between food and drug classifications can make it difficult to include health claims about flavonoids in product marketing [342]. These regulatory bodies require substantial evidence of efficacy and safety before allowing flavonoid-related health claims in dietary supplements [367]. Therefore, a collaborative approach among researchers, clinicians, industry stakeholders, and regulatory bodies is essential for the scientific advancement and acceptance of flavonoids as nutraceuticals.

Furthermore, ongoing research on the pharmacokinetics of flavonoids and their mechanisms of action will be crucial to establish more robust guidelines for their clinical application, to ensure that practitioners can responsibly recommend these compounds for health purposes [328].

### 6.1. Growth and Applications of Flavonoids

In recent years, the demand for flavonoids-based dietary supplements and functional foods has increased, due in part to their potential antioxidant, anti-inflammatory, and antitumor activity [368]. Among these is quercetin, one of the most studied flavonoids, which has been linked to improvements in several health parameters, increasing the demand [19,369]. Furthermore, plant-derived flavonoids, such as eriodictyol, are attracting considerable attention due to their beneficial health properties [370].

The rise in flavonoid supplementation is due not only to its effectiveness but also to changes in lifestyles and eating habits. Faced with the global increase in chronic diseases associated with poor diet and sedentary lifestyles, consumers are actively looking for preventive measures that incorporate nutraceuticals into their daily routines [371]. Research indicates that flavonoid supplementation can improve both athletic performance and overall health by mitigating exercise-associated oxidative stress [343]. Furthermore, it could potentially contribute to improved cognitive function thanks to its antioxidant activities [372].

Table 14 shows some applications of flavonoids in human health.

### 6.2. Market Dynamics and Consumer Trends

The global nutraceutical market is highly influenced by the behaviors and preferences of both consumers and healthcare professionals, who increasingly prioritize the origin and safety of ingredients [373]. Regulatory frameworks applied to nutraceuticals can significantly influence market dynamics, with transparency of labeling and clinically proven efficacy being crucial to building consumer trust [358]. However, disparities in regulations between different countries could affect product availability and market penetration in various regions.

The nutraceutical sector encompasses various market segments, from dietary supplements to functional foods, with flavonoid-rich products incorporated into segments such as beverages, health foods and cosmetics [374]. In particular, the functional foods market is using the incorporation of flavonoids as a marketing strategy to attract health-conscious consumers [375]. Recent studies underscore the importance of marketing strategies in the dietary supplement sector as companies adapt to evolving market demands [376].

## 7. Discussion

Flavonoids and other polyphenols have been extensively reviewed. The most novel contribution of this research and its positioning relative to previous reviews lies in its integrated framework, which links (i) molecular mechanisms of action, (ii) bioavailability limitations, (iii) the strength and limitations of available clinical evidence, (iv) translational endpoints relevant to therapeutic and nutraceutical use, and (v) market significance. This approach aims to move beyond the common review format that lists compounds, sources, and reported effects in isolation. By explicitly connecting mechanistic claims with exposure limitations and clinically interpretable outcomes, it increases the contribution to research [377,378]. Unlike reviews focusing primarily on individual compounds, this article contributes to understanding how flavonoids operate within complex biological systems shaped by food matrixes, microbial biotransformation, and interindividual variability [359,379]. We also highlight the interactions between the microbiome and flavonoids as a key translation axis that could help explain heterogeneous responses and support more targeted diet intervention strategies [79,283].

As we have seen, plant-derived bioactive compounds have a fundamental role in preventive medicine and in the design of dietary interventions to reduce the burden of chronic diseases. Within this framework, polyphenols and flavonoids in particular stand out for their ability to modulate certain cross-cutting biological processes, especially oxidative stress and low-grade chronic inflammation, which contribute to the pathogenesis of cardiovascular, metabolic and neurodegenerative disorders, as well as certain types of cancer [12,13,14,15,72]. This perspective aligns with the paradigm of “food as therapeutic medicine,” where the development of functional foods emerges as a pragmatic way to translate acquired knowledge into a more natural impact on at-risk populations [380,381].

The diet sources of polyphenols are heterogeneous and their actual contribution depends on the fraction consumed (pulp, skin, seeds, or peel) and on agronomic and technological variables that can increase or decrease their concentration [31,104]. In fruits, berries are recognized as exceptional reservoirs of anthocyanins and other flavonoids, with concentrations increasing during fruit development and reaching a maximum at full maturity [38,76]. Similarly, blueberries, blackberries, and strawberries have high levels of cyanidin-3-O-glucoside and related anthocyanins [106,382]. In grapes (*Vitis vinifera* L.), compartmentalization is relevant, with anthocyanins predominating in the fruit peel, while catechins and proanthocyanidins are found in the seeds, and resveratrol is distributed in both the peel and pulp. Furthermore, the profile varies according to the cultivar, and the pomace retains polyphenols of interest for extraction and valorization [57,107,108,109,110]. In citrus fruits, the peel contains higher levels of polyphenols than the pulp, and processing byproducts constitute strategic raw materials to recover hesperidin, naringenin, and other flavonoids [61,111,112].

At the molecular level, polyphenols exert antioxidant effects through complementary mechanisms. The first level involves the direct elimination of free radicals and ROS by donation of electrons or hydrogen atoms from phenolic hydroxyl groups, which depends on electron density and structural motifs such as catechols (e.g., hydroxytyrosol) [8,85]. The second level consists of enhancing endogenous defenses by increasing the activity or expression of antioxidant enzymes (superoxide dismutase, catalase, and glutathione peroxidase) and activating signaling pathways, as in the case of Nrf2, which reinforces the adaptive antioxidant response [383]. This dual mechanism, which involves both direct capture and defensive reprogramming, allows us to interpret the effects observed in vitro and in vivo and explains, to some extent, why even with moderate bioavailability, polyphenols can alter systemic redox tone when consumed consistently.

The anti-inflammatory component is expressed, in part, through suppression of pro-inflammatory cytokines and inhibition of signaling cascades associated with vascular and systemic inflammation. In particular, some polyphenols, such as hydroxytyrosol, have been described as reducing the expression of endothelial adhesion molecules and consequently leukocyte adhesion and transmigration. Similarly, the activation of anti-inflammatory pathways in immune and glial cells has been reported, with a reduction in mediators such as IL-6 and IL-8 [89,90,91]. Consistent with this, inhibition of NF-κB and modulation of the NLRP3 inflammasome described for flavonoids link inflammatory signaling with redox control and tissue protection [34,254].

Regulation of gene expression is another important dimension to consider. Resveratrol, which activates the SIRT1/PGC1α/SIRT3 axis, has implications for mitochondrial biogenesis, which is itself a stress response and metabolic regulation [62]. In parallel, modulation of Wnt/β-catenin by polyphenols has been linked in different studies to proliferation, differentiation, and ultimately neuroprotection [39].

In flavonoids, signaling pathways that are particularly relevant for vascular health have been identified. The PI3K-AKT pathway is a critical cascade through which flavonoids enhance endothelial function and thus reduce oxidative stress, thus promoting vasodilation and protecting against endothelial dysfunction [153,188,191]. Furthermore, flavonoids activate AMPK and are associated with the regulation of energy homeostasis and stress responses, while mechanistic studies have demonstrated inhibition of mTOR and associated pathways in the presence of flavonoid activators, supported by molecular coupling for the interaction of the AMPK ligand [94,192]. Finally, the increase in NO bioavailability is integrated as a functional mechanism to maintain vascular homeostasis and, therefore, to reduce blood pressure, with evidence of increased plasma NO after ingestion of flavonoid-rich fruits and vegetables [184,186,194]. Flavonoids modulate vasoactive factors, such as angiotensin-converting enzyme activity and endothelial signaling. Due to their antioxidant properties, they mitigate a central determinant of hypertension: oxidative stress associated with endothelial dysfunction [186,191,215]. In association studies, higher dietary flavonoid intake is inversely associated with hypertension, which would support a protective effect in at-risk populations [217].

The interest in specific flavonoids is based on some mechanistic evidence and their presence in relatively accessible foods such as pomegranate (*Punica granatum*), where the peel, in particular, concentrates anthocyanins, quercetin and catechins, which have antioxidant properties and potential anticancer effects [137,384]. Onion (*Allium cepa*), like pomegranate, is a highly valued source of quercetin and kaempferol. Quercetin in onions is associated with antioxidant capacity and modulation of inflammatory pathways and cell survival [140,141]. At the molecular level, quercetin exhibits antioxidant and anti-inflammatory activity, with effects on mediators such as IL-1β and IL-6, while kaempferol has been linked to the induction of apoptosis and the inhibition of tumor proliferation in models of some cancers [139,148]. Competitive inhibition of ENPP1 by quercetin and myricetin further increases the potential for insulin resistance and metabolic inflammation [147].

An interesting cross-cutting challenge for polyphenols is oral bioavailability, as their limited solubility, rapid metabolism, or intestinal instability can restrict systemic exposure. This point is particularly relevant when moving from whole foods to concentrated ingredients or supplements. In response, delivery systems have been developed that aim to increase dispersibility, potentially protecting against degradation, and thus promoting better absorption. These include nanoemulsions/micelles, liposomes, phytosomes, lipid or polymeric nanoparticles, and inclusion complexes with cyclodextrins. These systems have shown marked improvements in bioavailability in preclinical and clinical models for compounds such as curcumin, and their co-administration with enhancers such as piperine has markedly increased the exposure to curcumin in humans, although special caution is required due to potential drug interactions [385].

The bioavailability of polyphenols varies from plant to plant. In cruciferous vegetables, the growing conditions determine the accumulation of phenolic compounds, and drought-adapted varieties have been found to accumulate even higher levels, opening opportunities for cultivation strategies aimed at maximizing the functional density of the food [114]. In beverages, camellia tea (*Crotonin sinensis*) is an important source of catechins and flavonoids, with marked differences depending on whether it comes from green or black tea. This is due to the type of processing (minimal oxidation in green tea versus fermentation/oxidation in black tea), which determines the predominance of catechins over theaflavins and thearubigins [115,116,117,118,119]. On the other hand, coffee is notable for its chlorogenic acid content, which depends on the variety, roasting method, and brewing method [120,121]. For its part, red wine represents a complex matrix in which fermentation generates additional compounds by polymerization and condensation [122,123].

Consequently, the development of functional foods with plant bioactives requires the integration of criteria for stability, bioavailability, content standardization, and efficacy validation. Fermentation has been proposed as an innovative approach to improve bioactivity by modifying matrices and releasing potentially more available forms of antioxidant compounds, particularly in polyphenol-rich plant foods [386].

Regarding translation, critical gaps remain that must be addressed rigorously, given the marked heterogeneity of study designs, the insufficient duration of interventions, the variability in matrices and dosages, and the lack of standardization in the chemical characterization of extracts [387]. Consequently, it is necessary to strengthen clinical trials by increasing their scale and duration, incorporating mechanistic biomarkers in humans, and moving towards personalized nutritional standards. In this respect, it is essential to consider the genetic variability and microbiota of each individual, which influence both the metabolism and bioactivation of polyphenols. At the same time, regulatory and quality control frameworks must support the development of innovative ingredients, particularly when using encapsulation systems or bioavailability enhancers.

Furthermore, in the reviewed literature, bioavailability emerges as a key factor in efficacy, influenced by chemical structure, food matrix, digestion and metabolism, particle size, and microbiota-driven biotransformation [388]. These factors help explain why in vitro antioxidant capacity does not consistently predict in vivo outcomes and why formulation strategies (including encapsulation) can substantially alter exposure profiles [389,390]. Consistent with this, clinical evidence suggests the potential benefits of flavonoid-rich interventions on cognitive/neuroprotective outcomes, modulation of cardiometabolic risk, and inflammatory/immunometabolic endpoints [391,392]. However, comparability is limited by heterogeneity in populations, doses, matrices, duration of intervention, and outcome selection [393]. In parallel, the interactions of flavonoids with dietary components, supplements, and drugs (including metal chelation, redox activity, microbiome-dependent effects and modulation of metabolic pathways) represent both an opportunity for synergy and a source of variability and safety considerations in translation [394,395].

The relevance of nutraceuticals and the regulatory/market context, analyzed from an implementation perspective, establish that flavonoids represent an important nutraceutical category, but clinical translation depends on standardization (composition, stability, bioavailability), evidence-based claims, and regulatory alignment [396]. Market expansion is likely to be driven by demand for preventive health and improved formulations, but long-term credibility will depend on rigorous characterization, transparent labeling, and human evidence supporting clinically relevant endpoints.

In other words, polyphenols, with their ability to modulate oxidative stress, inflammation, and endothelial signaling, along with opportunities for formulation, fermentation, and sustainable recovery of byproducts, support their potential in prevention and therapeutic support. However, the real impact will depend on integrating mechanical and clinical evidence with standardization, safety, and sustainability, avoiding simplistic extrapolations. To the extent that technological innovation remains aligned with scientific validation and robust regulatory frameworks, it will be possible to move toward food and nutraceutical solutions that tangibly contribute to the reduction in chronic diseases [72,380,381].

This article synthesizes antioxidant chemistry (HAT/SET and metal chelation), redox-sensitive signaling, and mitochondrial quality control within an evidence-weighted framework that explicitly incorporates limitations, contradictions, and the bioavailability gate that governs in vivo exposure. By aligning mechanistic claims with metabolite predominance, dose realism, and clinically interpretable endpoints, the manuscript provides a decision-relevant basis for preventive and therapeutic hypotheses rather than a purely descriptive compilation.

Future work should prioritize standardized exposure and metabolite profiling, harmonized outcome selection in human trials, and stratification strategies accounting for microbiome-driven interindividual variability to enable more reproducible translation. This would allow for a more reliable translation into dietary guidelines and nutraceutical development with more rigorous clinical data.

## 8. Conclusions

Plant polyphenols constitute a class of chemically diverse and biologically versatile bioactive substances whose relevance to human health transcends their conventional designation as dietary antioxidants. Among phenolic acids, flavonoids, stilbenes, lignans, and other complex phenolic compounds, shared structural features (phenolic hydroxyl groups, catechol motifs, and conjugated aromatic systems) allow direct redox interactions and higher-order regulation of cell signaling. This article consolidates these dimensions into a unified perspective. That is, the health value of plant bioactives is determined by the convergence of molecular mechanisms, dietary context, processing history, and delivery to target tissues.

A key lesson that emerges is that bioavailability often limits biological potential. Low water solubility, chemical instability, extensive first-pass metabolism, and matrix-dependent release can significantly limit the fraction of an oral dose that reaches relevant targets in its active form. Therefore, bioavailability should be considered a fundamental scientific variable and a prerequisite for reliable translation. Encapsulation and stabilization technologies, such as nanoemulsions, liposomes, phytosomes, solid lipid or polymeric nanoparticles, inclusion complexes, and matrix-based delivery strategies, can protect labile molecules. They can also improve dispersibility and align release kinetics with physiological absorption processes, thereby increasing the likelihood of achieving measurable and reproducible effects.

This review offers a novel framework by comprehensively examining various studies, including the translational mechanisms linking antioxidant chemistry and signaling with bioavailability limitations, clinical evidence, and translational endpoints for therapeutic and nutraceutical applications. The available human data supports the potential benefits of flavonoid-rich interventions in the cognitive, cardiometabolic, and inflammatory domains. However, the heterogeneity of study designs and the variability in exposure limit the possibility of making definitive recommendations.

This research stimulates interest in future analyses that should prioritize well-characterized interventions, metabolite-based pharmacokinetics, standardized clinical endpoints, and a comprehensive assessment of dietary and drug-nutritional interactions. It also aims to improve the validation of clinical studies through the use of artificial intelligence and regulatory frameworks. This would allow for a more reliable translation into dietary guidelines and the development of nutraceuticals based on more rigorous clinical data.

In summary, this article aims to provide a coherent framework that connects structural classification, molecular mechanisms, dietary sources, and processing determinants, as well as technologies that improve bioavailability, within a preventive health therapeutic strategy. The main conclusion is that the “efficacy of polyphenols” is best understood as a systemic property determined by chemistry, matrices, metabolism and interindividual variability, rather than as an intrinsic attribute of an isolated compound.

## 9. Some Challenges and Future Prospects

Table 15 summarizes some challenges that could limit the application of polyphenols in more widespread therapeutic applications and outlines some priorities for future research to address them. These include barriers related to bioavailability, standardization, safety, interindividual variability, stability during processing, industrial scalability, and even the use of artificial intelligence to optimize extraction.

## Figures and Tables

**Table 1:** Classification of the main types of polyphenols, structural characteristics, and dietary sources.

Polyphenol Class	Description	Examples and Sources	References
Phenolic acids	Simple, non-flavonoid, low molecular weight polyphenols with an aromatic ring substituted with at least one carboxylic acid group. They are classified as benzoic acid derivatives (C_6_–C_1_) and cinnamic acid derivatives (C_6_–C_3_). They contribute significantly to the total antioxidant capacity of the diet and to the modulation of oxidative stress by neutralizing free radicals.	Hydroxybenzoic acids: gallic acid, protocatechiic acid, present in fruits, tea, and some seeds. Hydroxycinnamic acids: caffeic, ferulic, p-coumaric acids, and chlorogenic acid (the ester of caffeic acid and quinic acid), abundant in coffee, whole grains, fruits, and vegetables. Spices such as turmeric contain curcuminoids (curcumin and related compounds), which are structurally related due to their high density of phenolic groups.	[8,39,40,41,42,43,44,45,46]
Flavonoids	The most abundant subclass of polyphenols in nature, representing 60% of total polyphenols. They are characterized by a C_6_–C_3_–C_6_ skeleton composed of two aromatic rings (A and B) connected by a three-carbon chain that forms a heterocyclic ring (C). Variations in the degree of oxidation and in the substitution patterns (hydroxyls, methoxyls, sugars) give rise to different subclasses (flavonols, flavones, flavanones, flavan-3-ols, anthocyanins, isoflavones). They are generally found in the form of glycosides that require intestinal hydrolysis or hydrolysis by the intestinal microbiota to release the bioactive aglycone. They possess antioxidant, anti-inflammatory, and photoprotective properties.	Flavonols: quercetin, kaempferol, myricetin, present in onions, kale, lettuce, spinach, berries, and tea. Flavones: apigenin and luteolin present in parsley, celery, and chamomile. Flavan-3-ols (catechins and epicatechins) and their polymers (proanthocyanidins) present in green and black tea, cocoa and grape seeds. Flavanones: hesperidin and naringenin in citrus fruits. Delphinidin glycosides, responsible for the red, purple, and blue colors of blackberries, blueberries, strawberries, and grapes. Isoflavones: genistein and daidzein, abundant in soybeans and legumes, with phytoestrogenic activity.	[47,48,49,50,51,52,53,54,55]
Stilbenes	Non-flavonoid polyphenols characterized by a C_6_–C_2_–C_6_ skeleton, composed of two phenolic rings joined by an ethylene bridge. The geometric configuration (trans-or cis) modulates their stability and biological activity. They are notable for their cardioprotective, antioxidant, anti-inflammatory, and cell signaling pathway modulation potential.	Resveratrol (3,5,4’-trihydroxy-transstilbene), present in red grapes, red wine, blueberries, peanuts, and Japanese polygonaceae. Eterostilbene (trans-3,5-dimethoxy-4’-hydroxystilbene), a methylated derivative of resveratrol, which is found mainly in blueberries and in the heartwood of Pterocarpus marsupium. Resveratrol has been associated with the so-called “French paradox,” which links moderate red wine consumption with a lower incidence of cardiovascular disease.	[56,57,58,59,60,61,62,63,64,65,66,67,68,69]
Lignans	Polyphenols derived from the phenylpropanoid pathway, consisting of two units of phenylpropane (C_6_–C_3_) joined through their central carbons. In the human intestine, they are transformed by the microbiota into enterolignans (enterodiol, enterolactone) with phytoestrogenic activity and potential cardioprotective and metabolic effects.	They are found predominantly in flax and sesame seeds as well as in whole grains. Lignans such as pinoresinol and acetoxypinoresinol have been described in olive oil. The main enterolignans of intestinal origin are enterodiol and enterolactone, derived from secoisolariciresinol and other plant precursors.	[39,40,70,71]
Tannins	Heterogeneous group of high molecular weight polyphenols capable of forming complexes and precipitating proteins. They are divided into hydrolyzable tannins (esters of sugars with gallic or ellagic acid) and condensed tannins or proanthocyanidins (polymers of flavan-3-ol units). They possess a high antioxidant capacity, astringent effects, and antimicrobial activity.	Proanthocyanidins derived from catechins and epicatechins in grapes, red wine, cocoa, and grape seeds. Tannins are also present in tea, nuts, and various fruits, where they contribute to the sensory astringency and oxidative stability of foods.	[8,12,48,58,59,60,72]

**Table 2:** Limitations and contradictions in evidence of mitophagy with resveratrol-SIRT1.

Test Problem/Limitation	Scientific Interpretation	Implications for Data Interpretation	References
Substrate dependence of SIRT1 activation	Direct activation of SIRT1 by resveratrol depends on the experimental substrates and the assay conditions, leading to inconsistent results between studies.	Resveratrol should be described as a context-dependent modulator rather than a universal direct activator of SIRT1.	[79,83]
Indirect signaling mechanisms	Resveratrol can indirectly influence SIRT1-related pathways through AMPK activation and modulation of NAD^+^ metabolism.	SIRT1 can arise from network-level regulation rather than direct enzyme activation.	[80,81]
Complexity of mitophagy regulation	The regulation of PINK1/Parkin signaling and autophagy flux varies according to models, doses, and time points, resulting in heterogeneous findings.	Interpretation must distinguish between marker expression and validated autophagy flux measurements.	[82]

**Table 3:** Molecular mechanisms of action of polyphenols.

Main Mechanism	Biological Target Level	Description of the Mechanism	Examples of Polyphenols Involved	References
Antioxidant mechanisms	Direct elimination of ROS and free radicals	Polyphenols act as direct scavengers of free radicals and ROS by donating electrons or hydrogen atoms from their phenolic hydroxyl groups. The presence of catechol motifs and dopamine-like structures increases the efficiency of radical scavenging.	Hydroxytyrosol and other polyphenols with catechol fraction. Phenolic compounds with multiple aromatic OH groups.	[8,85]
Antioxidant mechanisms	Endogenous (enzymatic) antioxidant systems	Polyphenols improve the activity of endogenous antioxidant enzymes such as SOD, catalase, and glutathione peroxidase. This effect is exerted in part through the activation of the Nrf2 signaling pathway, which induces the expression of genes with antioxidant response elements (AREs).	Various flavonoids and non-flavonoid polyphenols that activate Nrf2 and regulate the transcription of antioxidant enzymes.	[86]
Antioxidant mechanisms	Flavonoids act primarily at the cellular level by interrupting oxidative chain reactions and strengthening endogenous defenses. They chelate transition metals and eliminate reactive oxygen species (ROS); for example, quercetin can stabilize iron and thus reduce Fenton-type reactions.	Metal chelation (Fe^2+^/Cu^2+^): the binding of transition metals limits the formation of metal-catalyzed ROS, including Fenton-type reactions. Elimination of free radicals: direct neutralization by electron transfer (SET) and/or hydrogen atom transfer (HAT), reducing oxidative damage. SET readings: the reduction capacity is commonly captured by FRAP and ABTS type assays.	Quercetin: abundant in apples and onions; chelates metals and eliminates free radicals. Kaempferol: present in fruits and vegetables; reduces oxidative stress by neutralizing free radicals. Luteolin: exhibits antioxidant and anti-inflammatory actions; activates chelation and detoxification pathways.	[87,88,89]
Anti-inflammatory mechanisms	Modulation of cytokine and endothelial adhesion molecules	Polyphenols reduce the production of pro-inflammatory cytokines and inhibit inflammatory signaling cascades. Hydroxytyrosol decreases the expression of VCAM-1, ICAM-1, and E-selectin in endothelial cells, thus reducing the adhesion of monocytes and lymphocytes.	Hydroxytyrosol and related phenolic compounds from olive oil.	[90,91]
Anti-inflammatory mechanisms	TREM2 in microglial cells	Polyphenols activate the TREM2-dependent anti-inflammatory pathway (trigger receptor expressed in myeloid cells 2) in microglia, reducing the release of pro-inflammatory cytokines such as IL-6, IL-8, IP-10 and RANTES.	Polyphenols with the ability to signal through TREM2 in microglia (including oleic compounds).	[90]
Modulation of gene expression	SIRT1/PGC1 Pathway α/SIRT3 and mitochondrial biogenesis	Resveratrol activates the SIRT1/PGC1α/SIRT3 pathway, promoting mitochondrial biogenesis and function. This pathway is a key in the stress response and metabolic regulation.	Resveratrol and other stilbenes capable of activating sirtuins.	[62]
Modulation of gene expression	Wnt/β-catenin	Wnt/β-catenin, which is involved in cell proliferation, differentiation, and neuroprotection. Regulation of this pathway may contribute to neuroprotective effects and preservation of tissue integrity.	Flavonoids and other polyphenols that interact with components of the Wnt/β-catenin pathway.	[39]
Modulation of gene expression	Genetic regulation in fibroblasts and tissue regeneration	Hydroxytyrosol and related polyphenols in olive oil positively regulate genes involved in fibroblast cell proliferation and differentiation, promoting wound healing and tissue regeneration.	Hydroxytyrosol and phenols from olive oil (including secoiridoid derivatives).	[92,93]
Function and autophagy	Activation of autophagy and the cellular stress response	Hydroxytyrosol induces autophagy by inhibiting histone deacetylases 1 and 2 (HDAC1/2), contributing to an improved stress response and metabolic homeostasis. This mechanism is particularly relevant in the cases of liver injury and metabolic diseases.	Hydroxytyrosol and other polyphenols with HDAC activity.	[94]

**Table 4:** Dietary sources of polyphenols, the predominant compounds and the factors that modulate their content.

Food Group/Matrix	Specific Examples	Main Polyphenols	Factors that Modulate the Content	References
Overview	Plant-based foods (fruits, vegetables, beverages, oils, spices, legumes and seeds)	Mixture of phenolic acids, flavonoids (flavonols, flavanones, flavan-3-ols, anthocyanins), lignans, and other non-flavonoid polyphenols.	Type of food, fraction consumed (pulp, skin, seeds, shell), growing conditions, extraction and processing	[31]
Fruits and vegetables (general)	Fresh fruits and vegetables	Phenolic acids, flavonoids (flavonols, flavanones), anthocyanins, proanthocyanidins	Cultivar, harvest maturity, environmental conditions, post-harvest handling, and type of processing.	[104]
Berries	Blueberries, blackberries, strawberries, and other berries.	Anthocyanins (e.g., cyanidin-3-O-glucoside) and other flavonoids	The phenolic content increases during fruit development; it reaches its peak at full maturity; it is influenced by the crop and the growing conditions.	[52,105,106]
Grapes and by-products	Grapes (Vitis vinifera), skins, seeds, and grape pomace	Anthocyanins (skin), catechins, and proanthocyanidins (seeds), resveratrol (skin and pulp)	Cultivar, degree of ripeness, cultivation practices, and winemaking technology; grape pomace retains a high content of polyphenols after the production of juice or wine.	[57,58,106,107,108,109,110]
Citrus fruits	Oranges, lemons, grapefruits, tangerines; pulp and peel	Flavanones (hesperidin, naringenin) and other flavonoids	Species and cultivars; higher concentration of polyphenols in the peel than in the pulp; influence of industrial citrus processing	[58,111,112]
Cruciferous and leafy vegetables	Broccoli, spinach, artichokes and other Mediterranean cruciferous vegetables	Quercetin and other flavonoids; also, glucosinolates, anthocyanins, and carotenoids.	Growing conditions; Mediterranean varieties adapted to drought show a higher accumulation of polyphenols than cultivars in environments with greater water availability	[113,114]
Plant-based beverages: Green tea	Infusions of minimally oxidized Camellia sinensis leaves	Catechins (EGCG and others) and other flavonoids	Minimal oxidation during processing; scalding and drying conditions; and infusion time and temperature.	[115,116]
Plant-based beverages: Black tea	Fermented/oxidized camellia sinensis leaf infusions	Theaflavins, thearubigins, and polymerized derivatives of catechins	Fermentation and oxidation of leaves; technological differences compared to green tea (boiling vs. fermentation); processing and infusion parameters.	[117,118,119]
Plant-based beverages: Coffee	Filter coffee, espresso, and other brewing methods.	Chlorogenic acid and other related phenolic acids	Bean variety, roast level, brewing method (filter, espresso, French press, etc.)	[8,120,121]
Plant-based drinks: red wine	Red wine made with red grapes	Anthocyanins, catechins, quercetin, resveratrol, and other proanthocyanidins	Winemaking process (maceration, fermentation, and aging); oxidative polymerization and condensation reactions during fermentation.	[122,123]
Vegetable oils: EVOO	Extra virgin olive oil	Hydroxytyrosol, tyrosol, and secoiridoid derivatives, in addition to other minor phenols	Olive variety, harvest time (early harvest oils have higher levels of polyphenols), extraction techniques, storage, and exposure to oxygen and light.	[124,125,126,127,128]
Products: Olive pomace	Olive pomace (solid residue after oil extraction)	Hydroxytyrosol, tyrosol, and other remaining phenolic compounds	Olive variety, oil extraction conditions, technologies applied to pomace processing (drying, solid–liquid extraction, etc.)	[129,130,131]

**Table 5:** Some rich sources of flavonoids, main compounds, mechanisms, and health benefits.

Source/Category	Predominant Flavonoids	Main Mechanisms of Action	Health Benefits	References
Pomegranate (*Punica granatum*) and by-products	Anthocyanins, quercetin, catechins, ellagitannins, and other polyphenols	Antioxidant and anti-inflammatory activity; modulation of signaling pathways involved in apoptosis and cell proliferation; regulation of mechanisms associated with lipid metabolism and insulin sensitivity.	Antioxidant properties and possible anticancer effects; reduction in inflammation; improvement of arterial function; potential positive impact on metabolic syndromes by modulating lipid metabolism and insulin sensitivity.	[137,138,139]
Onion (*Allium cepa*), especially the peel	Quercetin, kaempferol (flavonoids)	Powerful antioxidant activity; modulation of biochemical pathways related to inflammation and cell survival.	Protection against chronic diseases, including heart disease and cancer, through anti-inflammatory and antioxidant effects.	[140,141]
Blueberries (*Vaccinium* spp.)	Anthocyanins (a subclass of flavonoids) and other flavonoids	Biosynthesis of flavonoids regulated by proteins and plant transcription factors that promote their accumulation; potent antioxidant activity with neutralization of free radicals.	Reduction in oxidative stress and inflammation; potential decrease in the risk of cardiovascular and neurodegenerative diseases.	[142]
Citrus fruits (oranges, lemons, grapefruits)	Hesperidin, naringenin, quercetin, and other citrus flavonoids	Anti-inflammatory, antimicrobial and antioxidant properties; activation of the Nrf2 pathway, which regulates the expression of antioxidant genes; modulation of blood glucose levels.	Significant contribution to cardiovascular health; beneficial effects in the management of diabetes by reducing blood glucose and mitigating oxidative stress.	[143,144,145]
Quercetin (specific flavonol)	Quercetin (present in pomegranate, onion, citrus fruits and other plant sources)	Anti-inflammatory and antioxidant activity; modulation of intracellular signaling pathways; inhibition of pro-inflammatory mediators such as IL-1β and IL-6; competitive inhibition of ENPP1 (along with myricetin), a regulator of metabolic pathways related to insulin resistance and inflammation.	Chemopreventive and cardioprotective; potential in the management of metabolic syndromes (including diabetes) by attenuating inflammation and improving insulin sensitivity.	[139,146,147]
Kaempferol (specific flavonol)	Kaempferol (present in pomegranate, onion, and other vegetables)	Induction of apoptosis in malignant cells; inhibition of signaling pathways related to tumor proliferation.	Anticancer and chemopreventive, contributing to the reduction in tumor proliferation.	[148]
Myricetin and other flavonoids associated with ENPP1	Myricetin and structurally related flavonoids	Competitive inhibition of ectonucleotide pyrophosphatase/phosphodiesterase 1 (ENPP1), a key regulator in metabolic pathways related to insulin resistance and inflammation.	Potential application in the management of metabolic syndromes, including diabetes, by improving insulin signaling and reducing inflammatory processes.	[147]
Flavonoids and epigenetic modulation	Quercetin, kaempferol, luteolin, and other flavonoids	Modulation of gene expression through epigenetic mechanisms, including changes in miRNA profiles related to inflammation and immune response.	Possible improvement of diseases ranging from allergies to cancer by adjusting genetic and immune networks.	[149]
Summary: Wide range of health benefits	Dietary flavonoids (quercetin, kaempferol, catechins, anthocyanins, hesperidin, naringenin, among others)	Combined antioxidant, anti-inflammatory, chemopreventive, and modulator of cell signaling and epigenetics.	Prevention and possible management of chronic diseases (cancer, metabolic syndrome, type II diabetes, cardiovascular diseases, chronic inflammatory processes); the bioavailability and effective action of these flavonoids are critical for their therapeutic impact.	[150,151]

**Table 6:** Synthesis of the strength of evidence in the main mechanisms of polyphenols with limitations and contradictions.

Mechanism	Typical Biological Assessment Criteria	Strength of Evidence (In Vitro/Animal/Human)	General Force	Key Limitations/Contradictions
Direct radical scavenging (HAT/SET) and reducing capacity (FRAP/ABTS type tests)	Chemical antioxidant capacity; ROS cell probes; lipid and protein oxidation markers	High/Low–Moderate/Low	Low–Moderate	Dependence on test conditions; supraphysiological dose in vitro; metabolites predominate in vivo; ROS probes may be nonspecific
Metal chelation (Fe^2+^/Cu^2+^) and inhibition of metal-catalyzed oxidation	Chelation constants; inhibition of Fenton-type reactions; markers of oxidative damage	High/Moderate/Low	Moderate	Competition with endogenous ligands/proteins; pH and matrix effects; limited validation of human biomarkers
Induction of endogenous defenses (Keap1–Nrf2/ARE)	of Nrf2; ARE-driven genes (e.g., HO-1, NQO1, GCLC); antioxidant enzyme activity	Moderate/Moderate/Low–Moderate	Moderate	Cell and tissue-specific responses; time/dose (hormesis); inconsistent enzyme endpoints between models
Modulation of anti-inflammatory signaling (e.g., NF-κB/cytokines; endothelial adhesion markers)	Cytokine panels; NF-κB activity; adhesion molecules (VCAM-1/ICAM-1); inflammatory biomarkers	Moderate/Moderate/Low	Low–Moderate	Heterogeneous models and assessment criteria; mixing effects; confounding factors in human studies; limited standardized clinical outcomes
Mitochondrial homeostasis and quality control (AMPK–mTOR; SIRT1/PGC-1α; mitophagy)	Markers of mitochondrial respiration/biogenesis; mitophagy reporters (PINK1/Parkin); autophagy flux	Moderate/Low–Moderate/Low	Low	Assay dependence and context sensitivity; conflicting findings for direct SIRT1 activation; Dose realism and tissue exposure
Assessment of the variability of dietary sources and exposure	Estimated intake; variability in food composition; exposure based on biomarkers (when available)	Moderate (observational); Low–Moderate (intervention)	Moderate (association)/Low–Moderate (causal)	Residual confounding and measurement error; cultivar/region/processing variability; difficulty in attributing effects to individual subclasses

**Table 7:** Medicinal plant species associated with the improvement of enzyme antioxidant defenses (SOD, catalase, and GPX).

Species (Scientific Name)	Common Name	Plant Bioactives	Associated Enzymatic Defenses (SOD/CAT/GPX)
*Camellia sinensis*	Green tea/Tea	Catechins (e.g., EGCG)	SOD, CAT, GPX
*Curcuma longa*	Turmeric	Curcuminoids (curcumin)	SOD, CAT, GPX
*Vitis vinifera*	Grape	Stilbenes (resveratrol) and flavan-3-oles	SOD, CAT, GPX
*Ginkgo biloba*	Ginkgo	Flavonoids; terpenoids (ginkgolides, bilobalides)	SOD, CAT, GPX
*Panax ginseng*	Ginseng	Saponins (ginsenosides)	SOD, CAT, GPX
*Rosmarinus officinalis*	Rosemary	Rosmarinic acid; phenolic diterpenes (carnosol/carnosic acid)	SOD, CAT, GPX
*Allium sativum*	Garlic	Sulfur compounds (allicin; S-allylcysteine)	SOD, CAT, GPX
*Ginger officinale*	Ginger	Gingerols and shogaols	SOD, CAT, GPX
*Olea europaea*	Olive/Olive	Secoiridoids (oleuropein); phenolic alcohols (hydroxytyrosol, tyrosol)	SOD, CAT, GPX
*Withania, sleep aid*	Ashwagandha	Withanolides (withaferin A and related)	SOD, CAT, GPX

**Table 8:** Key limitations and contradictions affecting the interpretation of the antioxidant mechanisms analyzed in this section.

Issue	Key Limitations	Contradictions/Variability	Implications for Interpretation/Reporting Needs
Concepts and definitions of oxidative stress	The definitions are consistent, but the mechanistic framework often merges radicals and non-radicals into a single category; biomarkers are not interchangeable across different studies.	Different studies operationalize oxidative stress using different endpoints (e.g., MDA, 8-OHdG, protein carbonyls, antioxidant enzyme activity), which produces non-comparable effect sizes.	Specify which biomarkers are used and why; avoid treating single marker as definitive evidence of modulation of oxidative stress.
Oxidative stress and pathogenesis of chronic diseases	Oxidative stress is frequently described as a common pathway, but causality is difficult to infer because oxidative markers may be subsequent consequences rather than primary drivers.	The strength of the association varies depending on the context, stage, and tissue of the disease; interventions that improve oxidative markers do not always translate into clinical outcomes.	Framing oxidative stress as a contributing mechanism; distinguishing biomarker enhancement from disease modification; prioritizing studies with clinically relevant results or validated surrogate endpoints.
Direct elimination of radicals/ROS	Many in vitro assays (e.g., DPPH/ABTS/FRAP) reflect chemical reduction capacity under non-physiological conditions and do not capture metabolism, protein binding, or cell compartmentalization.	A strong antioxidant capacity in vitro may coexist with weak or inconsistent in vivo effects due to low exposure and rapid conjugation; different radical/assays may classify compounds differently.	Avoid equating the potency of the chemical assay with the biological efficacy; report realistic concentrations, consideration of metabolites, and cellular/animal validation when available.
Positive regulation of endogenous antioxidant defenses	Changes in enzyme activity may reflect adaptive responses to stress rather than direct protection; tissue specificity and timing (acute vs. chronic) influence the direction and magnitude of the effects.	Some studies report an increase in SOD/GPx/CAT, while others report no change or bidirectional effects depending on the dose, model, and initial redox state.	Interpret enzyme induction in context (dose, duration, tissue); include pathway-level evidence (e.g., Nrf2 targets, glutathione status) and recognize dose-dependent hormesis.

**Table 9:** Molecular pathways and signaling mechanisms modulated by flavonoids.

Pathway/Mechanism	Primary Biological Function	Description of the Effect of Flavonoids	Examples/Featured Compounds	References
PI3K-AKT	Endothelial function, vasodilation, and oxidative stress control	The PI3K-AKT pathway constitutes a signaling cascade through which flavonoids improve endothelial function and reduce oxidative stress. Activation of this pathway is associated with improved endothelium-dependent vasodilation, increased cell survival, and reduced oxidative damage, which generally contributes to cardiovascular protection.	The diet flavonoids present in fruits and vegetables modulate PI3K-AKT and promote vasodilation and vascular health.	[153,188,191]
AMPK	Metabolic sensor and regulator of energy homeostasis	Flavonoids activate AMP-activated protein kinase (AMPK), a crucial metabolic sensor involved in regulating energy homeostasis and the cellular stress response. Homoplantaginin activates AMPK and inhibits phosphorylation of mTOR, p70S6K, and TFEB; these effects are attenuated by the AMPK inhibitor (Compound C). Molecular coupling studies show a favorable interaction between homoplantaginin and the AMPK protein.	Homoplantaginin and other flavonoids capable of activating AMPK and modulating mTOR/p70S6K/TFEB signaling.	[192,193]
Bioavailability of nitric oxide (NO)	Endothelial function, vasodilation, and regulation of blood pressure	Flavonoids improve endothelial function by increasing the bioavailability of nitric oxide (NO), a key molecule for vasodilation and maintenance of vascular homeostasis. Increased NO availability improves vascular function and reduces blood pressure. Consumption of fruits and vegetables rich in flavonoids has been associated with increases in plasma NO levels and improvements in vascular function.	Flavonoids present in fruits and vegetables (e.g., flavonols, flavanones, and other subtypes that promote NO synthesis and bioavailability).	[184,186,194]

**Table 10:** Flavonoids, regulation of blood pressure and related cardiovascular conditions.

Cardiovascular Condition	Main Mechanisms	Physiological/Clinical Effects	Behavior	Types of Flavonoids and Plant Sources (Examples)	References
Regulation of blood pressure	Modulation of vasoactive factors (including angiotensin-converting enzyme) and endothelial signaling; antioxidant properties that reduce oxidative stress; increased NO bioavailability and vasodilation.	Maintenance of vascular homeostasis and contribution to normalization of blood pressure; reduction in hypertension by improving endothelial function and decreasing oxidative damage.	The diet flavonoids of fruits and vegetables act on ACE, the endothelium, and NO production.	Flavanones: naringenin/hesperidin—citrus fruits (orange, grapefruit, lemon). Flavan-3-ols (flavanols): catechins/epicatechins (incl. EGCG): green/black tea; cocoa. Flavonols: quercetin/kaempferol: onion (especially red), kale, broccoli, spinach. Anthocyanins: cyanidin and derivatives: berries (blueberry, blackberry, strawberry), red grapes, pomegranate.	[186,191,215,216,217]
Atherosclerosis	Modulation of oxidative stress and inflammation through multiple molecular pathways; inhibition of key pathways involved in the progression of atherosclerosis; vascular anti-inflammatory action.	Prevention and slowing of the progression of atherosclerosis; reduction in vascular inflammation and endothelial damage associated with plaque formation.	Naringenin decreases endothelial infiltration of monocytes/macrophages and vascular inflammation; natural products of plant origin that target inflammation as a therapeutic approach.	Flavanones: naringenin: citrus fruits (grapefruit, orange). Flavan-3-ols: catechin/expetichin: tea; cocoa; grape seeds/skin. Flavonols: quercetin: onion, kale, broccoli, apple. Anthocyanins: berries and red grapes (high content in the skin).	[218,219,220,221,222,223]
Hypertension	Antioxidant properties that mitigate oxidative stress (a central contributor to endothelial dysfunction and hypertension); improvement of endothelial function by increasing NO bioavailability, vasodilation, and preservation of vascular homeostasis.	Antihypertensive effects mediated by reduction in oxidative stress, improvement of vasoreactivity, and endothelial protection; lower risk of hypertension associated with increased intake of flavonoids.	Association studies show that the dietary intake of flavonoids is inversely related to hypertension in American adults.	Flavanones: hesperidin/naringenin: citrus fruits (orange, lemon, grapefruit). Flavan-3-ols: catechins/epicatechins: tea and cocoa. Flavonols: quercetin/kaempferol: onion, kale, broccoli, spinach. Anthocyanins: berries and red grapes.	[11,186,191,216]
Vascular inflammation	Inhibition of vascular inflammatory signaling, including modulation of NF-κB, inhibition of inflammasome activation, and reduction in pro-inflammatory cytokine production.	Reduction in vascular inflammation, a key pathological process in cardiovascular disease; substantial contribution to the cardioprotective properties of flavonoids.	Reduction in pro-inflammatory cytokines and inflammatory mediators that perpetuate inflammation of the vascular wall.	Flavonols: quercetin/kaempferol: onion, kale, broccoli, spinach. Flavan-3-ols: EGCG/catechins: green/black tea; cocoa. Flavones: apigenin/luteolin: parsley, celery, chamomile. Anthocyanins: berries, red grapes, pomegranate.	[224,225]
Endothelial dysfunction and vascular health	State characterized by imbalance between vasodilation and vasoconstriction, increased ROS, inflammatory responses, platelet aggregation, autophagy, and apoptosis; endothelial activation with increased inflammatory mediators and cell adhesion molecules.	It contributes critically to the pathogenesis of multiple cardiovascular diseases; it promotes the adhesion, rolling, and transmigration of leukocytes, perpetuating vascular inflammation.	The endothelium acts as a key component of the vascular system and the inflammatory response; preserving its integrity is essential for cardiovascular health.	Flavan-3-ols: epicatechin/catechins: cocoa; tea. Flavanones: hesperidin/naringenin: citrus fruits. Flavonols: quercetin; onion, kale, broccoli. Anthocyanins: berries, red grapes.	[191,226,227,228,229,230,231]

**Table 11:** Cross-sectional limitations and contradictions in the evidence base of polyphenols in disease prevention and therapeutic applications.

Disease Section/Area	Key Limitations	Contradictions/Sources of Heterogeneity	Implications for Interpretation and Future Perspectives
Cardiovascular diseases	Heterogeneity of interventions (food vs. supplements; variable doses and duration) and frequent dependence on indirect outcome measures (volume of fever, blood pressure, lipids). Characterization of polyphenols and metabolites is often incomplete, limiting comparability.	The effects in different trials and cohorts are inconsistent, probably reflecting differences in food matrices, baseline risk, and concomitant medication; null results are also reported.	Interpret mechanistic plausibility independently of the magnitude of the clinical effect. Future studies should standardize exposure characterization, use validated biomarkers of intake/metabolism, and extend duration with predefined cardiovascular endpoints.
Metabolic diseases and diabetes	Many studies are preclinical or short-term, and the doses used in vitro and in animals can exceed the circulating concentrations achievable in humans. Human trials are often small and heterogeneous in terms of endpoints (fasting glucose, insulin, HbA1c) and baseline diet.	The reported effects on insulin sensitivity and glycemic control vary depending on the baseline phenotype, the context of the intestinal microbiome, and the formulation. The benefits observed for specific compounds are not generalizable to different classes of polyphenols.	Stratify by metabolic phenotype and medication status and prioritize clinically relevant endpoints and dose–response designs. Whenever possible, link results to measured metabolites and mechanisms, rather than relying solely on inferred intake.
Neurodegenerative diseases	Translational interpretation is limited by uncertainty about brain exposure, transport across the blood–brain barrier, and the long latency of neurodegenerative outcomes. Clinical evidence remains comparatively limited and endpoints differ between studies.	The strong neuroprotective signals observed in cell models may not be translated to in vivo because conjugated metabolites predominate in systemic exposure, and neuronal targets may require sustained dosing. Observational associations can be influenced by diet and overall lifestyle.	Emphasize pharmacokinetics and metabolite-driven mechanisms and use biomarker-based assays (oxidative stress, neuroinflammation, mitochondrial markers) with appropriate duration and cognitive or functional results.
Cancer prevention and therapeutic potential	Many anticancer mechanisms are derived from in vitro studies at concentrations that cannot be achieved through diet. The antioxidant versus pro-oxidant behavior depends on the context (cell type, redox state, metal availability), and clinical evidence is limited.	The epidemiological findings are mixed and some interventions show no effect. In oncology, antioxidant activity can have a double effect if it interferes with therapies based on oxidative mechanisms.	Anticancer claims should be framed conservatively, distinguishing between prevention and adjuvant therapy. Future work should prioritize physiologically relevant dosing, tumor context specificity, and safety and interaction assessments, along with mechanism of action.
Inflammatory and autoimmune diseases	Most of the evidence is mechanical or preclinical, with few statistically powerful clinical trials. The heterogeneity of the disease, the variability in endpoints, and the limitations in bioavailability make comparisons between studies and compounds difficult.	Inhibition of the observed pathway in vitro (e.g., modulation of NF-κB or inflammasome) may not be reproduced in vivo due to dose, metabolism and tissue distribution. Therefore, clinical signals vary depending on the indication and patient population.	Use standardized inflammatory biomarkers and clinically meaningful outcomes, and consider patient stratification (disease activity, immunomodulatory therapy). Connect the proposed mechanisms with the measured exposure and interaction with the target.

**Table 12:** Representative clinical evidence on dietary flavonoids in the main outcome domains.

Domain/Health Context	Type of Study	Population	Intervention (Source)	Key Results	References
Cognition/neuroprotection	Randomized double-blind trial	Older adults with mild cognitive impairment	Grape and blueberry extract rich in polyphenols	Improved cognitive outcomes vs. control	[264]
Cognition/neuroprotection	Synthesis of clinical evidence (systematic review)	Mixed adults; varies by trial	Higher intake of flavonoids/foods rich in flavonoids	RCTs report significant cognitive improvements; heterogeneity persists	[266,267]
Neurotrophic signaling	Narrative clinical evidence (nutraceutical approach)	It varies depending on the studies included.	Nutraceuticals rich in polyphenols	Reported increases in BDNF/CREB activity associated with neuronal function	[265]
Glycemic control (risk of type 2 diabetes)	Human intervention study	healthy adults	Flavonoids from raspberry leaf tea	Improved glycemic/insulinemic responses	[268]
NAFLD risk	Cohort evidence	population cohorts	The diet intake of anthocyanins	A higher intake of anthocyanins is associated with a lower risk of NAFLD.	[269,270]
CVD risk factors	Meta-analysis	Populations at risk	Anthocyanins/flavonols (dietary)	It improves lipid profiles and blood pressure.	[271]
CVD + glycemic markers	Randomized controlled trials	Adults (varies)	Dark chocolate, green tea (foods rich in flavonoids)	Improved glycemic control and cardiovascular risk markers	[272,273]
Inflammation/immune function	Synthesis of clinical evidence	It varies depending on the condition	Dietary flavonoids/polyphenols	Potential immunomodulation; reduction in markers of chronic inflammation	[274]
Atopic dermatitis	Dietary supplementation (clinical)	Patients with atopic dermatitis	Polyphenol supplementation	Reduced inflammatory markers	[275]
Overweight/metabolic inflammation	Evidence of human intervention	Overweight populations	Pomegranate juice (rich in flavonoids)	Beneficial effects on metabolic and inflammatory markers; modulation of the microbiota	[276]
Metabolic/inflammatory results	Human studies	It varies	citrus flavonoids	Results of positive metabolic control and inflammatory response	[277]

**Table 13:** Translational synthesis of contributions, limitations, and contradictions across evidence levels and application contexts.

Focus Area	Key Contribution	Limitations/Contradictions/Sources of Heterogeneity
Bioavailability as a determinant of in vivo efficacy	The therapeutic relevance is framed as limited by exposure: efficacy depends on bioavailability and pharmacokinetics, not just on in vitro antioxidant capacity.	Many mechanistic studies use concentrations that cannot be achieved in vivo; the original compounds may be lower relative to the conjugated forms/metabolites, which complicates attribution.
Structure-matrix effects (glycosides vs. aglycones; solubility/stability)	Summarize how the chemical structure and food matrix govern solubility, stability, hydrolysis, and absorption (glycosides require hydrolysis; aglycones are often absorbed faster).	Not all subclasses behave similarly; processing can increase (release/fermentation) and decrease (degradation) bioavailability; the effects depend on the matrix and the class of flavonoids.
Digestion, bioaccessibility, and first-pass metabolism	Introduces bioaccessibility as a determining factor and details degradation during gastric/intestinal digestion; it highlights enzymatic interactions and first-pass metabolism.	In vitro digestion models differ (protocols, enzymes, bile), creating inconsistent stability estimates; circulating levels often reflect conjugates rather than original compounds.
Biotransformation and metabolites driven by the microbiota	The hydrolysis/fermentation of the microbiota and the generation of metabolites are emphasized as central to bioactivity; a concrete example (hesperidin aglycone) is provided.	Bidirectional interactions between the microbiome and flavonoids introduce interindividual variability; the bioactivity of the metabolites may exceed or differ from that of the original compounds, complicating claims about the mechanism.
Processing/particle size and preparation of extracts (without a section focused on extraction)	Retention of only the aspects relevant to the exposure: smaller particles/fermentation can increase release and absorption, thus favoring practical optimization.	Processing can also degrade flavonoids; results vary depending on the technique, temperature, oxygen exposure, and matrix; not all improvements translate into systemic exposure in humans.
Dietary interactions (co-ingestion of fats; matrix synergy)	Highlights dietary interactions (e.g., fats facilitate the absorption of fat-soluble flavonoids; fibers/probiotics improve bioaccessibility).	The effects of interaction depend on the dose, timing and dietary patterns; co-ingestion varies between trials, reducing comparability and reproducibility.
Drug-nutrient interactions and safety in polypharmacy	Integrates clinically relevant interaction risks: modulation of CYP450, transporters, and altered pharmacokinetics of co-administered drugs.	The direction and magnitude of CYP effects vary depending on the flavonoid, formulation, and dose; supplement-level exposures may differ from dietary intake; clinical evidence may be limited for specific drug classes.
Synthesis of clinical evidence across all domains	Provides a structured synthesis based on domains (cognition/neuroprotection, cardiometabolic risk, inflammation/immunity) and points out design-related limitations.	Heterogeneity in populations, doses, matrices, duration, and assessment criteria limits comparability; some tests are associative (cohort/meta-analysis) rather than causal; selection of outcomes varies widely.
Encapsulation and stabilization to improve exposure.	It positions encapsulation (liposomes/nanoparticles, spray drying, maltodextrin, and lyophilization) as a pragmatic strategy to improve stability/bioaccessibility and potentially pharmacokinetics.	Many encapsulation studies are based on in vitro assays; the improvement in stability does not always translate into clinically significant results; variability in carriers and characterization methods limits comparisons between studies.

**Table 14:** Many health applications of flavonoids.

Health Application/Use Case	Main Rationale/Claimed Function	Illustrative Flavonoids/Product Context	Representative Outcomes	References
Dietary supplements & functional foods (general)	Increased demand driven by reported antioxidant, anti-inflammatory, and antitumor activity.	Flavonoid-based dietary supplements; functional foods enriched with flavonoid.	Growing consumer uptake for preventive health routines and mitigation of chronic-disease risk.	[368,371]
General health support (commonly studied flavonoid)	Flavonoids associated with improvements in multiple health parameters.	Quercetin (highly studied; common in supplement products).	Linked to improvements in health parameters, supporting increased demand.	[19,369]
Broad health promotion (emerging plant flavonoids)	Interest in structurally distinct plant flavonoids with beneficial health qualities.	Eriodictyol (plant-derived flavonoid; emerging nutraceutical interest).	Attracting attention to beneficial health qualities (specific endpoints depend on the study context).	[370]
Sports performance/exercise recovery	Mitigation of exercise-associated oxidative stress as a mechanism of performance and health benefits.	Flavonoid supplementation in athletic/active populations.	Reported improvements in athletic performance and overall health by reducing oxidative stress associated with exercise.	[343]
Cognitive function support	Potential contribution to improved cognitive function, plausibly through antioxidant-related mechanisms.	Flavonoid supplementation; flavonoid-rich dietary patterns/products.	Potential improvements in cognitive function (endpoint definitions vary between studies).	[372]

**Table 15:** Main challenges and future prospects for the therapeutic and industrial application of polyphenols.

Area/Challenge	Main Limitation	Future Perspectives and Course of Action	References
Improved bioavailability and therapeutic application	Inherently low bioavailability makes it difficult to achieve the systemic concentrations necessary for therapeutic efficacy.	Develop strategies to increase bioavailability through advanced encapsulation, chemical modification, and biotechnological synthesis.	[397]
Standardization and marketing	Marketing requires confirming the safety profile and demonstrating efficacy through clinical trials.	Strengthen safety assessment, standardization of formulations, and design/execution of robust clinical trials.	[251]
Individual variation in response	Interindividual variability in metabolism and response to polyphenol supplementation.	Incorporate microbiota/genetic stratification, response biomarkers, and personalized nutrition approaches.	[261]
Optimization and standardization	The extraction efficiency varies according to the source of the plant, the target compounds, and the operating conditions, making comparability and implementation difficult.	Optimize parameters per matrix/composite and standardize protocols to facilitate comparative analysis and industrial adoption.	[398]
Degradation and preservation of compounds	Thermal degradation, oxidation, and hydrolysis during extraction/processing reduce bioactivity.	Develop extraction and stabilization strategies that minimize degradation and maximize recovery efficiency and bioactivity.	[397]
Optimization, scalability, and industrial implementation	Gap between laboratory-scale performance and industrial adoption due to equipment costs, operational complexity, and scale limitations.	Addressing costs/benefits, simplifying operations, validating scalability, and designing robust industrial processes.	[399]
Neural networks and machine learning for extraction	Traditional models may be insufficient for complex nonlinear relationships in extraction processes.	Apply RNA/ML to predict and optimize performance; integrate with assisted extraction (e.g., enzymatic + ultrasound) to obtain more accurate predictive models.	[400]

## Data Availability

No new data was created or analyzed in this study. Data sharing is not applicable to this article.
